# Dentate gyrus ensembles gate context-dependent neural states and memory retrieval

**DOI:** 10.1126/sciadv.adn9815

**Published:** 2024-08-02

**Authors:** Cesar A.O. Coelho, Andrew J. Mocle, Alex D. Jacob, Adam I. Ramsaran, Asim J. Rashid, Stefan Köhler, Sheena A. Josselyn, Paul W. Frankland

**Affiliations:** ^1^ Program in Neurosciences and Mental Health, The Hospital for Sick Children, Toronto, ON, Canada.; ^2^ Department of Physiology, University of Toronto, Toronto, ON, Canada.; ^3^ Department of Psychology, University of Toronto, Toronto, ON, Canada.; ^4^ Department of Psychology, University of Western Ontario, London, ON, Canada.; ^5^ Institute of Medical Sciences, University of Toronto, Toronto, ON, Canada.; ^6^ Child & Brain Development Program, Canadian Institute for Advanced Research, Toronto, ON, Canada.

## Abstract

Memories of events are linked to the contexts in which they were encoded. This contextual linking ensures enhanced access to those memories that are most relevant to the context at hand, including specific associations that were previously learned in that context. This principle, referred to as encoding specificity, predicts that context-specific neural states should bias retrieval of particular associations over others, potentially allowing for the disambiguation of retrieval cues that may have multiple associations or meanings. Using a context-odor paired associate learning paradigm in mice, here, we show that chemogenetic manipulation of dentate gyrus ensembles corresponding to specific contexts reinstates context-specific neural states in downstream CA1 and biases retrieval toward context-specific associations.

## Teaser

Dentate gyrus context ensembles constrain memory search to context-appropriate associations.

## INTRODUCTION

The recall of specific past events relies on an interplay between retrieval cues and underlying memory traces (or engrams) (*1*–*4*). However, a retrieval cue may acquire multiple associations and, as a consequence, retrieval is less efficient when many cue associations need to be parsed to identify the target memory (*5*, *6*). Sensitivity to context is one way in which our brains increase the efficiency of retrieval induced by a cue with multiple associated memories. The same cue (e.g., the sound of a gun firing) may have very different associations that can be disambiguated by context (e.g., being in a bad area of town late at night versus at the starter’s blocks at a running track). In other words, context constrains memory search, prioritizing access to those memories associated with the context at hand.

Empirical evidence supporting contextual modulation of retrieval comes from behavioral and imaging experiments in humans and nonhuman animals. Behaviorally, matching encoding and retrieval contexts boosts retrieval success (*4*, *7*–*12*). In imaging studies, the degree of overlap between encoding and retrieval neural states predicts retrieval success (*8*, *12*–*14*), particularly when the environmental context is the same (*15*). However, these studies are correlational in nature (*16*) and do not provide direct evidence that context-dependent neural states bias which cue-associations are retrieved in a particular situation. Here, we combine ensemble activity tagging and chemogenetic methods to manipulate context-dependent neural states in mice. We test whether manipulation of these states alters downstream neural dynamics and biases memory retrieval to context-specific content. To do this, we used a context-odor paired association task (*17*–*19*) in which odor cues acquire conflicting positive and negative associations whose meaning can only be disambiguated by context.

## RESULTS

### Mice use context to disambiguate the meaning of odor cues with conflicting associations

**Fig. 1.**
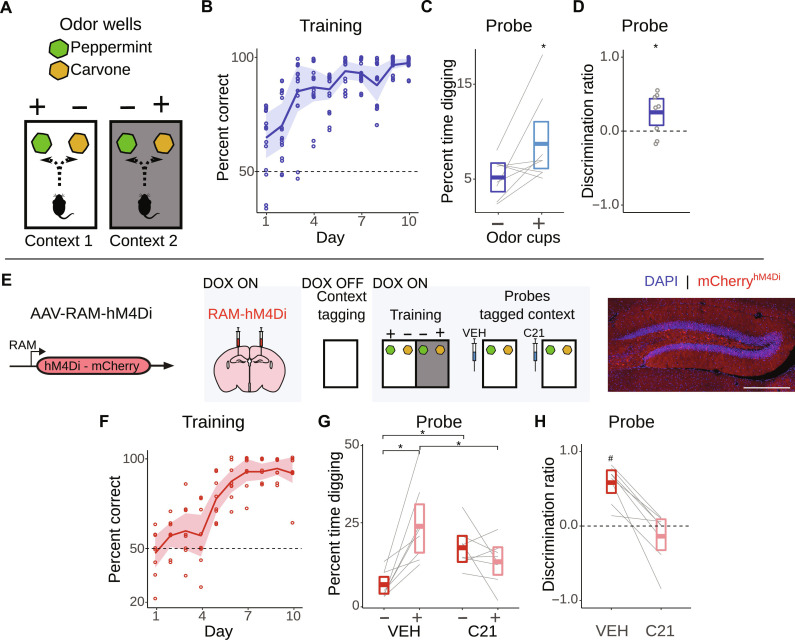
Disambiguation of cue associations depends on DG context ensembles. (**A**) Context-odor paired associate task. Mice (*N* = 8) were trained concurrently in two spatial contexts. In context 1, digging in peppermint-scented bedding was reinforced, whereas in context 2, digging in carvone-scented bedding was reinforced. (**B**) Performance improved over training days [shading represents 95% confidence interval (CI)]. (**C**) Percent time spent digging and (**D**) discrimination score in probe test after training (box represents 95% CI; dashed line represents chance performance). (**E**) Left: AAV-RAM-hM4Di was microinjected into the DG. Middle: Removal of doxycycline (DOX) permitted hM4Di tagging of DG neuronal ensemble active during pre-exposure to one of the to-be-discriminated contexts. After training, mice (*N* = 8) were probed in a tagged context after either vehicle (VEH) or C21 treatment. Right: hM4Di-expressing (red) neurons in DG [blue = 4′,6-diamidino-2-phenylindole (DAPI)]. Scale bar, 500 μm. (**F**) Performance improved across training (shading represents 95% CI). (**G**) Percent time spent digging and (**H**) discrimination score in probe test after training (box represents 95% CI; dashed line represents chance performance). *P < 0.05 (group comparison); ^#^*P* < 0.05 (comparison to zero).

In the discriminative context-odor paired association task, mice were trained concurrently in two spatial contexts (black context and white context). In one context, digging in one well (scented with peppermint), but not another well (scented with carvone), was reinforced with a sugar pellet reward. In the other context, these contingencies were reversed, with digging in the carvone-scented well, but not the peppermint-scented bedding well, being reinforced (Fig. 1A). Performance in this task improved over the course of training, with mice reaching >90% accuracy by the end of training (Fig. 1B and table S1; see Supplementary Text for details of statistical analyses). One day following training, memory was evaluated in unreinforced probe tests in the two contexts. In these tests, mice spent more time digging in the well containing the previously reinforced odor, indicating that mice learned to use the context to disambiguate the meaning of the odor cues, access the correct memory, and display context-appropriate behavior (Fig. 1, C and D, and table S2).

### Odor cue disambiguation depends on context-tagged dentate gyrus ensembles

The hippocampus plays an essential role in representing context (*20*–*27*). To ask whether neuronal representations of to-be-discriminated contexts in the hippocampus are essential for disambiguating the meaning of the odor cues in this task, we used an adeno-associated virus (AAV)–based, doxycycline (DOX)–dependent RAM (robust activity marking) ensemble activity tagging method (Fig. 1E and fig. S1) (*28*). This permitted expression of the inhibitory designer receptors exclusively activated by designer drugs (DREADD), hM4Di, selectively in neuronal ensembles in the dentate gyrus (DG) of the hippocampus that were activated during initial exposure to one of the two contexts. Following training (Fig. 1F and table S3), mice were tested twice in the tagged context and, before each test, treated with either the DREADD ligand, C21, or VEH (vehicle; order counterbalanced). VEH-treated mice spent more time digging in the well containing previously reinforced odor in that tagged context. However, chemogenetic silencing of DG “context” ensembles abolished this bias. These mice dug equally in either well (Fig. 1, G and H, and tables S4 to S6), indicating that tagged context ensembles in the DG are necessary for disambiguating the meaning of the odor cues in this task.

### Activation of tagged dentate gyrus ensembles biases memory retrieval in a context-dependent manner

**Fig. 2.**
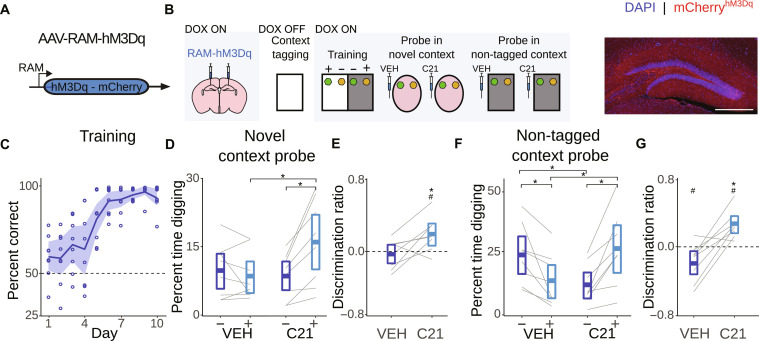
Activation of dentate gyrus context ensembles reinstates context-specific memory retrieval. (**A**) AAV-RAM-hM3Dq was microinjected into DG. (**B**) Left: Removal of DOX permitted hM3Dq tagging of DG neuronal ensemble active during pre-exposure to one of the to-be-discriminated contexts. After training, memory was probed in a novel context or the non-tagged context after either VEH or C21 treatment. Right: hM3Dq-expressing (red) neurons in the dentate gyrus (blue = DAPI). Scale bar, 500 μm. (**C**) Performance improved across training (shading represents 95% CI) (*N* = 8). (**D**) Percent time spent digging and (**E**) discrimination score in probe test in the novel context. (**F**) Percent time spent digging and (**G**) discrimination score in probe test in the non-tagged context (box represents 95% CI; dashed line represents chance performance). *P < 0.05 (group comparison); ^#^*P* < 0.05 (comparison to zero).

We next asked whether the reactivation of tagged context ensembles in the DG would be sufficient to bias retrieval towards associations learnt in that context. We used the RAM tagging system to express the excitatory DREADD, hM3Dq, in DG granule neuronal ensembles that were activated by exploration of one of the to-be-discriminated contexts (Fig. 2, A and B). Following training (Fig. 2C and table S7), mice were placed in a novel context containing two wells with the previously sampled scents. In this ambiguous situation, VEH-treated mice spent equivalent time digging in either well. In contrast, C21-treated mice spent more time digging in the scented well corresponding to the tagged context (Fig. 2, D and E, and tables S8 to S10), indicating that reactivation of DG neuronal ensembles representing a tagged context facilitated recall of the related odor cue memory.

One day later, these same mice were placed in the non-tagged context. In unreinforced probe tests, VEH-treated mice spent more time digging in the previously reinforced well, as expected. However, reactivation of the DG neuronal ensemble corresponding to the tagged context reversed this context-specific preference. C21-treated mice spent more time digging in the scented well that was previously reinforced in the tagged context rather than the current (non-tagged) context in which they were placed (Fig. 2, F and G, and tables S11 to S13). The same pattern of results was observed when activated DG granule cells were tagged with the excitatory opsin, ChR2, rather than chemogenetic actuators (see fig. S2 and tables S14 and S15 for validation of tagging approach using contextual fear conditioning). In the novel context, photostimulation of the tagged ensemble biased preference toward the scented well that was previously reinforced in the tagged context. Similarly, in the non-tagged context, photostimulation of tagged ensemble reversed preference, with mice exhibiting digging preference corresponding to the tagged (rather than current non-tagged) context. Optogenetic biasing of retrieval occurred when tagging took place during exposure to the context alone (fig. S3 and tables S16 to S24) or during a single context training session (fig. S4 and tables S25 to S29). These results suggest that reactivation of the DG context ensemble is sufficient to override sensory input from the external world in driving context-appropriate decision-making and allow internally generated patterns of activity to take on that role.

### Training alters CA1 population activity in a context-dependent manner

**Fig. 3.**
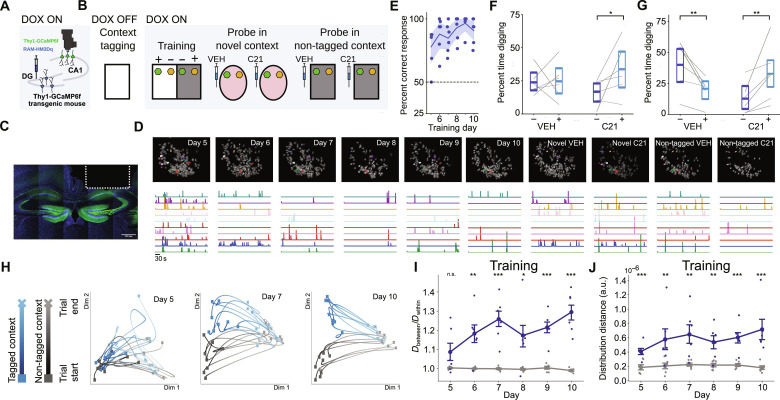
Experience-dependent plasticity of CA1 contextual representations. (**A** and **B**) AAV-RAM-hM3Dq was microinjected into DG in Thy1-GCaMP6f mice, and a miniature microscope was implanted above CA1. DOX removal permitted hM3Dq tagging of DG neuronal ensembles active during context pre-exposure. After training, memory was probed in novel and non-tagged contexts after either VEH or C21 treatment. (**C**) The dotted lines indicate a miniature microscope lens implanted above CA1 (blue = DAPI; green = GCaMP6f^+^ cells). Scale bar, 500 μm. (**D**) Example of CA1 imaging field across sessions. Randomly selected cells are colored by cell identity, and their corresponding denoised fluorescence traces are plotted below. (**E**) Performance improved across training (shading represents 95% CI) (*N* = 6). (**F** and **G**) After training, memory was probed in novel (F) and non-tagged (G) contexts. In both contexts, C21 treatment increased digging time in the well which was reinforced in the tagged context during training. (**H**) CA1 population activity structure across training revealed by dimensionality reduction (trajectories plotted on a reduced set of dimensions using Laplacian eigenmaps). (**I**) For each training day, the average cosine distance was computed between PVs of trials within the same context (*D*_within_) and between PVs of trials in different contexts (*D*_between_). PV distance between contexts increased relative to distance within a context across training (blue = actual). Gray lines indicate distance ratios with randomized context IDs (shuffle) (*N* = 6 mice). (**J**) Latent-space distance between PVs from different contexts increased with training. For each training day, PVs were projected to a five-dimensional space using principal components analysis, and the distance between the five-dimensional distributions for each context was calculated (blue = actual). Gray lines and dots indicate chance level distances calculated by randomizing PV context identity (shuffle) (*N* = 6 mice). a.u., arbitrary unit; n.s. not significant. *P < 0.05, **P < 0.01, and ***P < 0.001.

CA1 is downstream from the DG and is important for retrieving context-specific memory content through the process of pattern completion (*29*). Therefore, one possibility is that the reactivation of tagged context ensembles in the DG reinstates context-specific patterns of activity in downstream hippocampal regions, including CA1. Accordingly, we evaluated whether context-specific patterns of neural activity emerge in CA1 during training and whether manipulation of DG context ensembles is sufficient to reinstate features of those patterns during subsequent probe tests. In mice expressing a genetically encoded calcium indicator (Thy1-GCaMP6f mice), we implanted custom-built miniature fluorescence endoscopes (*30*) to image CA1 neuronal activity during training and probe sessions (Fig. 3, A to D; fig. S5, A to D; and tables S30 to S35). As before, we used the RAM system to express hM3Dq in DG neuronal ensembles that were active during exposure to one of the to-be-discriminated contexts following the removal of DOX. In probe tests following training (Fig. 3E and table S36), chemogenetic reactivation of these ensembles induced well preference corresponding to the tagged context in both the novel (Fig. 3F and tables S37 to S39) and the non-tagged contexts (Fig. 3G and tables S40 to S42), replicating our previous results. Consistent with the idea that the reactivation of tagged context ensembles in the DG helps disambiguate the meaning of odor cues, C21-treated mice exhibited shorter latency to dig when tested in the novel context (fig. S5E and tables S43 to S45).

When animals learn, neural dynamics increasingly reflect relevant task dimensions (*31*–*34*). To visualize how the dynamics of CA1 population activity differed along the critical task dimension of context, and how these differences evolved over the course of training, we used dimensionality reduction (*35*, *36*). In each context, population activity followed a distinct path through neural space. These neural trajectories became more differentiated over the course of training (Fig. 3H), suggesting that context-modulated neural states in CA1 diverged with continued learning. To quantify this divergence, we computed the ratio of the mean between-context versus within-context trial-averaged, population vector (PV) distance. This ratio increased with training (Fig. 3I and tables S46 and S47), consistent with the idea that neural states diverged along the critical task dimension (i.e., context). Similarly, we found the distance between PVs (unaveraged) in principal component (PC) space increased with training (Fig. 3J and tables S48 and S49). The differentiation of CA1 neural states was not confounded by features of mouse behavior (e.g., speed) (tables S50 and S51).

### Dentate gyrus ensemble reactivation does not alter global population dynamics in CA1

**Fig. 4.**
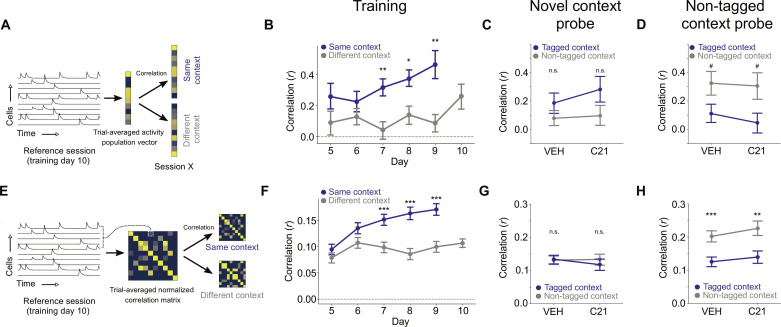
Activation of DG context ensemble does not reinstate long–time scale population activity features of CA1 context–specific neural states. (**A** and **B**) For each context and training day, PVs of average activity were calculated. Blue dots indicate the average similarity between a given day’s PV and the day 10 PV from the same context. Gray dots signify the similarity between a given day’s PV and the day 10 PV from the different contexts (*n* = 217 to 280 cells per session, *N* = 6 mice). (**C** and **D**) DG ensemble activation does not shift the population of active cells to a tagged context–like state in either (C) novel (*n* = 202 to 278 cells per session, *N* = 6 mice) or (D) non-tagged (*n* = 162 to 251 cells per session, *N* = 6 mice) contexts. PVs of average activity were calculated for all VEH and C21 trials, and their correlation to training day 10 PVs from the tagged (blue) and non-tagged (gray) contexts was calculated. (**E** and **F**) For each context and training day, a pairwise normalized correlation matrix (NCM) was computed (see Materials and Methods). Dots indicate average similarity between a given day’s NCM and the day 10 NCM from the same context (blue) or opposite context (gray) (*n* = 217 to 280 cells per session, *N* = 6 mice). (**G** and **H**) DG activation does not change the pattern of average pairwise functional connectivity in either (G) novel (*n* = 202 to 278 cells per session, *N* = 6 mice) or (H) non-tagged context (*n* = 162 to 251 cells per session, *N* = 6 mice). NCMs of average pairwise correlation were calculated for all VEH and C21 trials, and their correlation to training day 10 NCMs from the tagged (blue) and non-tagged (gray) contexts was calculated. ^#^*P* < 0.06, *P < 0.05, **P < 0.01, and ***P < 0.001.

To identify which features of CA1 population activity best differentiate the two contexts, we calculated PVs of average fluorescence for each cell across each trial (Fig. 4A). To isolate context-associated activity from scent- or digging-associated activity, we excluded neuronal activity when the mouse was digging or near either of the wells. Using the last day of training (day 10) as a reference, we compared PVs on day 10 to PVs from preceding days in both the same and different contexts. We found that PVs from the same contexts were more similar to one another compared to those from different contexts. Moreover, the distinction between the same and different contexts increased as a function of training despite no differences in the overlap of detected cells (Fig. 4B, fig. S6A, and tables S52 to S56), consistent with the idea that neural states in CA1 are differentiated by experience (*31*). In the subsequent probe tests in the novel and non-tagged contexts, we similarly calculated PVs for each trial in VEH or C21 conditions and compared them to the PVs from the tagged and non-tagged contexts on day 10 of training. C21 treatment did not increase PV similarity to the tagged context in either the novel or the non-tagged context probes (Fig. 4, C and D, and tables S57 and S58). This suggests that chemogenetic reactivation of DG ensembles does not shift overall CA1 population activity toward neural states associated with the tagged context.

We next investigated training-induced changes in global interactions among pairs of active CA1 neurons over an entire trial since this may more fully capture the statistical structure of the neural states that emerge through learning (*37*, *38*). To do this, we compared pairwise correlations from day 10 of training to each of the contexts for each of the preceding training days (Fig. 4, E to H, and tables S59 to S62). We found that the structure of pairwise interactions changed in a context- and experience-dependent manner across training. However, in the novel and reversal probe tests, C21 treatment did not shift the overall structure of interactions between all cell pairs toward the tagged context, suggesting that global changes across the entire CA1 population do not contribute to the expression of context-linked memories. Since CA1 neurons with adjacent place fields would be expected to show high pairwise correlations (*39*), this further suggests that chemogenetic reactivation of tagged context DG ensembles does not reinstate place maps of the associated context.

### Dentate gyrus ensemble reactivation reinstates context-specific ensemble activity in CA1

**Fig. 5.**
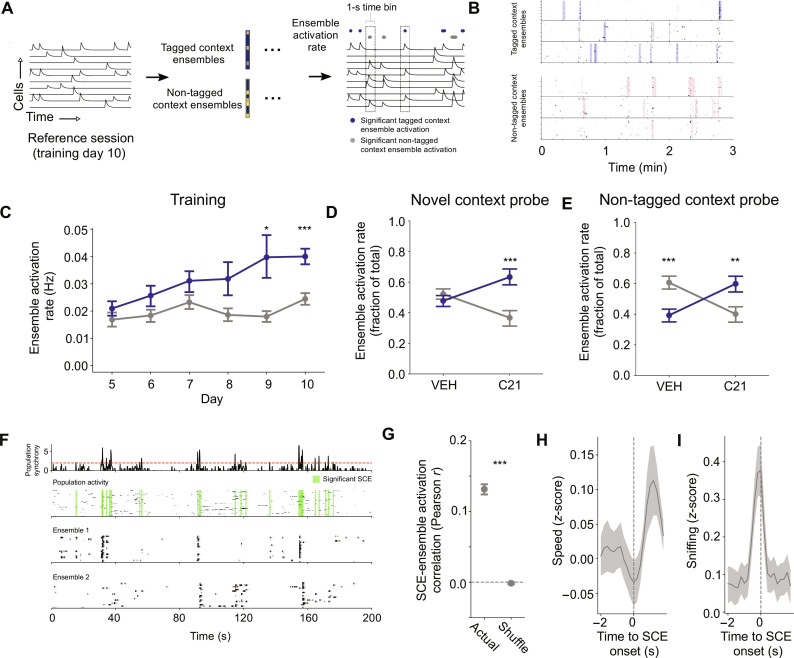
Activation of DG context ensemble reinstates CA1 context–specific ensemble activation events. (**A**) Measuring context and experience-dependent plasticity in ensemble activity. (**B**) Examples of detected ensembles from training day 10 in the tagged and non-tagged contexts. Highlighted regions indicate significant ensemble activations. (**C**) Context-dependent differences in ensemble activation rates over training. On training day 10, ensembles were extracted from population activity in each context. The activity of these ensembles was then plotted on previous training days (days 5 to 9). For each training day, blue dots represent the frequency of significant activations for ensembles that were active in the same context on day 10. Gray dots represent the frequency of significant activations in opposite-context ensembles (*n* = 576 trials, *N* = 6 mice). (**D** and **E**) DG ensemble activation increases the activation rate of ensembles extracted from the tagged context in the (D) novel (*n* = 57 trials, *N* = 5 mice) and (E) non-tagged contexts (*n* = 69 trials, *N* = 6 mice). Ensembles were extracted from population activity from either the tagged or non-tagged context on training day 10. For VEH and C21 trials, tagged context and non-tagged context ensemble activation rates were calculated and normalized to the total number of ensemble activations in the particular probe session. (**F**) Example of synchronous calcium events (SCEs) alongside ensemble activation events. Top row: Fraction of active cells across time. Second row: Population activity, with significant SCEs highlighted in green. Bottom two rows: Activity traces of two example ensembles showing activation preferentially during SCEs. (**G**) Ensemble activation events correlate with SCEs at above chance (shuffle) levels (*n* = 36 sessions, *N* = 6 mice). (**H** and **I**) Time course of (H) locomotion and (I) sniffing behavior relative to SCE onset, shaded regions indicate ±SEM. *P < 0.05, **P < 0.01, and ***P < 0.001.

Rather than changing global patterns of activity, an alternative possibility is that artificial reactivation of DG ensembles biases memory recall by reinstating context-specific and temporally discrete ensemble activity in CA1 (i.e., more specific dynamics between >2 CA1 neurons that occur in subsecond epochs within the trial). For ensemble analysis, we extracted coactive ensembles from each context on day 10 and computed the ensemble activation rate on each of the preceding days (Fig. 5, A and B). We found that ensemble occurrence changed in a context- and experience-dependent manner across training (Fig. 5C and tables S63 and S64). Moreover, in the probe tests, C21 treatment increased the relative reactivation frequency of tagged context ensembles, both in the novel context and in the non-tagged context (Fig. 5, D and E, and tables S65 to S68). This suggests that context-specific ensemble reactivation, rather than global changes across the entire CA1 population, contributes to the expression of context-linked memories. Consistent with this, chemogenetic reactivation did not alter general features of CA1 activity (figs. S5, B and C, and S6, B and C, and table S69).

One possibility is that global changes across the CA1 population (i.e., pairwise correlations) reflect hippocampal activity evoked by external cues (i.e., the current context), whereas discrete ensemble events reflect internally driven retrieval of tagged context-specific content. We found that ensemble events occurred preferentially during periods of synchronous population activity in CA1 (Fig. 5, F and G, and table S70), when the mouse was moving slowly and engaged in sniffing behavior (Fig. 5, H and I; fig. S6, D to F; and tables S71 to S73). Since sharp-wave ripples occur during periods of synchronous population activity in CA1 (*40*, *41*) and are linked to memory retrieval (*42*–*47*), this suggests that the observed temporally discrete ensemble activations represent sharp-wave ripple-associated memory recall events during moments of potential “deliberation.” To test the causal role of CA1 ensemble reactivation, future studies may use closed loop-based approaches to test whether interfering with ensemble expression impairs the retrieval of context-linked associations.

## DISCUSSION

Previous studies tagged DG ensembles active during contextual fear conditioning and established that ensemble reactivation leads to artificial memory recall, typically indexed by measuring freezing behavior in an otherwise safe context. However, in these studies, contexts were paired with only a single outcome (footshock). In contrast, in the current study, we trained mice in a cue disambiguation task, where cues are paired with conflicting outcomes in a context-dependent manner. We found that reactivation of DG context ensembles reinstated context-specific neural states in CA1 and biased retrieval toward context-specific content and allowed disambiguation of cues with conflicting, context-dependent associations.

Studies using observational methods (e.g., calcium imaging and electrophysiological recordings) have shown that natural memory retrieval is associated with spatially and temporally specific patterns or sequences of neural activity in the hippocampus (*42*, *48*, *49*). On the other hand, studies using interventional methods (e.g., ensemble tagging combined with optogenetics) have demonstrated that simultaneous activation of tagged hippocampal ensembles, especially in the DG, is sufficient to evoke behaviors that resemble natural recall (*21*, *23*, *50*). In our imaging experiments, we examined the impact of Gq-DREADD activation of context-tagged DG neurons on patterns of neural activity in CA1. Since DREADD-based activation does not impose any particular temporal structure, it is perhaps expected that reactivation of context-tagged DG ensembles reinstated context-specific ensemble activity in CA1 and reinstated context-appropriate digging behavior (both in the novel context and the non-tagged context).

However, we also found that optogenetic reactivation of context-tagged DG ensembles similarly reinstated context-appropriate digging behavior. This result is perhaps more unexpected given that optogenetic stimulation is highly structured (e.g., 20 Hz). It suggests that this temporally structured activation is transformed by the network into context-specific sequences in downstream regions (e.g., CA3 and CA1), which can then drive behavior. There is recent evidence suggesting that this occurs following the optogenetic activation of hippocampal engram neurons (*51*). Optogenetic stimulation of “place avoidance memory”–tagged hippocampal neurons induced retrieval of the associated memory and, during photostimulation, CA1 activity maintained intrinsic ensemble discharge relationships. In the current study, ensemble events preferentially occurred during sharp-wave ripples. Because sharp-wave ripples are initiated in CA3 (*52*–*54*), we speculate that the DG activation constrains which ensembles are recruited in CA3/CA1, and, in this way, biases retrieval.

In our experiments, the context alone, before training the mice on the odor associations, was tagged. Following training, reactivation of the tagged context ensemble facilitated recall of the related odor association. This indicates that, after initial tagging, subsequent experiences that occur in the same context become linked to the corresponding DG ensemble. This may occur via an updating mechanism (*55*) and is consistent with the view that the hippocampus binds contextual information with items (e.g., peppermint scent) and events (e.g., pairing peppermint scent with sugar reward) occurring in that context (*56*). Subsequently, the same item (i.e., peppermint scent) may become associated with a different outcome in another context (i.e., no reward). Accordingly, reactivated DG ensembles may serve as effective retrieval cues, allowing mice to access context-specific memory content, disambiguate the meaning of items with conflicting, context-dependent associations, and produce context-appropriate behavior. Viewed in this way, it might be reasonable to suggest that ensembles in the DG function as indices that coordinate memory retrieval (*57*–*59*) by constraining memory search to those memories that are most relevant for the context at hand. In the present study, DG ensembles were tagged by exposure to a physical context. A related possibility is that DG ensembles corresponding to other types of context (e.g., temporal) or nonphysical, internally generated neural states (e.g., mood) may also function to constrain retrieval in similar ways (*8*, *60*–*62*).

## MATERIALS AND METHODS

### Experimental subjects

In behavioral experiments, we used male and female wild-type (WT) mice that were the F1 offspring from a C57BL/6N x 129Svev cross. For calcium imaging, we used Thy1-GCaMP6f mice on a BL6J/129SvEv hybrid background. These mice were derived by crossing mice hemizygous for the Thy1-GCaMP6f transgene on a BL6J background (GP5.17 line, Jackson Laboratories) (*63*) with WT 129SvEv mice. Mice were weaned at 21 days, housed four to five mice per cage, maintained on a 12-hour light-dark cycle (lights on during the day), and given food and water ad libitum. All procedures were conducted in accordance with policies of the Hospital for Sick Children Animal Care and Use Committee (protocol #1000063648) and conformed to both the National Institutes of Health (NIH) Guidelines on Care and Use of Laboratory Animals and the Canadian Council for Animal Care (CCAC).

### Viral vectors

In the optogenetic experiments, we labeled active neuronal ensembles with AAV-DJ viral vectors made with plasmids as follows: pAAV-RAM-d2TTA::TRE-ChR2-WPREpA or pAAV-RAM-d2TTA::TRE-EGFP-WPREpA (#84471, Addgene, Watertown, MA; www.addgene.org/84471/, www.addgene.org/84469/). For the chemogenetic experiments, we made AAV-DJ viral vectors using DREADD cDNAs for hM3D(Gq)-mCherry and hM4D(Gi)-mCherry from the pAAV-CaMKIIa-hM3D(Gq)-mCherry and pAAV-CaMKIIa-hM4D(Gi)-mCherry plasmids, provided as a gift by B. Roth (#50476 and #50677, respectively, Addgene; https://n2t.net/addgene:50476, https://n2t.net/addgene:50477). These were subcloned into the multiple cloning site of the plasmid pAAV-RAM-d2TTA::TRE-MCS-WPREpA, provided as a gift by Y. Lin (# 63931, Addgene, Watertown, MA; www.addgene.org/63931/). The RAM system has the RAM promoter incorporated in the Tet-Off system, which allowed us to control green fluorescent protein (GFP), ChR2, hM4D, and hM3D expression by administration or withdrawal of DOX from the diet [ON DOX and OFF DOX, respectively (*28*)]. When OFF DOX, a synthetic activity-regulated promoter, comprising minimal AP-1, Fos, and Npas4 promoter sequences, tags active neurons (*28*).

The DOX diet (Cedarlane, Burlington, ON) consisted of pellets of regular chow with DOX. In preliminary experiments, mice expressing AAV-RAM-GFP were fed a DOX diet at different concentrations under free feeding [DOX (50 mg/kg)] or food restriction [DOX (200 mg/kg)] (as described below). Forty-eight hours following DOX removal, mice were fear-conditioned. Mice were perfused 24 hours later, and numbers of GFP^+^ nuclei in the DG were assessed. GFP tagging was only observed in the OFF DOX conditions at both DOX concentrations, indicating that this tagging system is not “leaky.” Because mice were food deprived in the context-odor paired associate task, we used the 200 mg/kg diet in all subsequent experiments (fig. S1).

### Surgery

#### Viral infections

Mice were pretreated with atropine sulfate (0.1 mg/kg, ip), anesthetized with isoflurane-oxygen mix (3% isoflurane for initial induction and 1 to 2.5% through nose cone thereafter), and administered the analgesic meloxicam (4 mg/kg, sc). Mice were placed in a stereotaxic frame, the scalp was incised, and holes were drilled above the DG bilaterally. The virus was microinjected into the DG [anterior-posterior (AP), −2.2 mm; medial-lateral (ML), ∓1.5 mm; dorsal-ventral (DV), −2.2 mm] using glass micropipettes connected by polyethylene tubing to a microsyringe (Hamilton, Reno, NV) mounted onto a pump (volume was 1.2 μl per hemisphere at a rate of 0.15 μl/min). All viral vectors were thawed and diluted 1:2 in VEH immediately before use. The micropipettes remained in place for 5 min after injections to ensure diffusion of solution. For optogenetic experiments, optrodes were implanted above the microinjection site (DV, −2.0 mm). Optrodes were fixed to the skull with three jeweler screws and black dental cement. Following microinjection/optrode implantation, the scalp was sutured and antibiotic cream was applied.

#### Lens implantation

Mice were pretreated with atropine sulfate (0.1 mg/kg, ip) and dexamethasone (5 mg/kg, ip) to reduce brain inflammation and swelling during surgery. Mice were anesthetized with an isoflurane-oxygen mix (3% isoflurane for initial induction and 1 to 2.5% through nose cone thereafter) and placed into a stereotaxic frame. Lenses for calcium imaging were implanted as previously described (*30*). A craniotomy was performed above the right CA1 (AP, −2.0 mm; ML, +1.5 mm), the dura was carefully removed, and cortical tissue was gently aspirated while continuously applying chilled artificial cerebrospinal fluid. A 2-mm-diameter gradient index lens (ILW-200-P0250-055-NC, GoFoton) was implanted at a depth of −1.5 mm relative to the skull surface and fixed to the skull using three jeweler screws and dental cement. After surgery, mice were treated with analgesics (meloxicam, 4 mg/kg, sc) and 1 ml of 0.9% saline (subcutaneous) to prevent dehydration. Mice recovered for 4 to 6 weeks before the start of imaging.

#### Optogenetic and chemogenetic ensemble manipulation

For the chemogenetic experiments, C21 [0.1 mg/kg diluted in phosphate-buffered saline (PBS), ip] or VEH was injected 1 hour before the start of the behavioral procedure. For photostimulation, we used a laser source (Laserglow Technologies, Toronto, ON) connected to a function generator (Keysight Technologies, Mississauga, ON) via optic fibers that plugged into the optrode implants. Photostimulation was conducted using blue light [473-nm wavelength, 15-ms pulses, 20 Hz, 1 to 2 mW, 5 Volts peak to peak (vpp), and 30% duty cycle]. During the “Light ON” test sessions of the context-odor pair associates task, photostimulation was delivered both during the pretrial period (~20 s) and throughout the trial. Photostimulation terminated at the end of each trial and remained off while the mouse was in the transport cage.

### Contextual fear conditioning

#### Behavioral apparatus

For contextual fear conditioning, we used two different chambers. The first chamber, context A (31 cm × 24 cm × 21 cm; Med Associates, St. Albans, VT), consisted of a front, top, and back wall made of transparent acrylic pieces, two side walls made of modular aluminum, and a shock grid floor composed of stainless steel rods (diameter, 3.2 mm; spaced 7.9 mm apart). Behavior was monitored via cameras mounted above the chamber [4 frames per second (fps)]. The second context, context B (32 × 25 × 25 cm, Med Associates), was partitioned into a semicircle by adding a white, Plexiglas arc-shaped insert. The front wall of the context was a transparent acrylic door, and the chamber was housed within a larger sound-attenuating chamber. Fans provided background noise (60 dB) and white house lights illuminated the chamber. Behavior was monitored by cameras in front of the chamber (30 fps). A near-infrared pass filter was used to avoid interference from the photostimulation light in the video recordings.

#### Behavioral procedure

After surgery, mice were maintained on a DOX diet and handled for 5 min/day for 3 days. Two days before training, DOX was removed from the diet. During training, mice were placed in context A for 120 s, and then five footshocks (0.5 mA, 1 s) were delivered, spaced 30 s apart. After the last shock, mice remained in the context for 60 s and then were returned to their home cage and placed back on a DOX diet. Two days later, mice were placed in context B for a total of 12 min. This 12-min test was divided into four blocks of 3 min, and photostimulation (Light ON) was applied in the second and fourth blocks. Freezing behavior, characterized by immobility except for breathing movements (*64*), was scored continuously by an experimenter blind to the experimental manipulations. For the training data, we calculated the percent time spent freezing in the “pre-shock” (0 to 120 s), “during shock” (120 to 240 s) and “post-shock” (240 to 300 s) periods. For test data, we calculated the percent time spent freezing in the combined light ON versus the combined light OFF blocks.

### Context-odor paired associate task

#### Behavioral apparatus

For the context-odor paired associate task, we used three contexts. Two of the contexts were rectangular in shape and made of acrylic walls and floor with dimensions 24.5 cm × 18 cm × 45 cm (width × height × depth). The first context (“white context”) was white, with smooth surfaces and floor. The second context (“black context”) had a textured black floor and smooth walls with black and white stripes. The third context (“novel context”) had an elliptical wall with dimensions 31 cm × 39 cm × 18 cm (shorter radius, longer radius, height) and all surfaces were covered with patterned brown wallpaper. Contexts were cleaned with water between trials for a given mouse and with 70% ethanol after each mouse. A camera connected to a computer was placed above the contexts and all sessions were recorded at 4 fps for offline behavioral scoring (LimeLight Software, Actimetrics, Wilmette, IL). For the imaging experiment, behavior was recorded at a higher framerate (20 fps).

In each context, wells filled with bedding and scented with different odors were positioned on the left or right, respectively. The wells comprised an outer container with two inserts. The outer container was adhered to the floor of the cage so that the mice could not “flip” the well. The middle insert contained a piece of paper with 100 μl of the odor solution. The inner insert rested above the middle insert containing the scented paper. The base of the inner insert was perforated (to allow the odor to percolate) and the insert was filled with bedding. In this study, we used carvone(−) and peppermint as odors, diluted in mineral oil at a 1:5 ratio. The odors were replenished before each mouse was tested.

To avoid the mice guiding themselves just by the sugar scent of the food reward, we replaced the bedding in the inner insert of the wells every four trials. We also placed ~5 sugar pellets in the middle insert (together with the odor source) for both the rewarded and non-rewarded odors. These were not accessible to the mouse and therefore ensured that mice could not use sugar scent to guide their choices.

#### Food restriction and shaping

Before the behavioral task, we restricted food availability until mice reached ~90% of their free-feed body weight. Mice remained at ~90% free-feed body weight for the remainder of the experiment. The mice usually reached 90% body weight within 6 days of food restriction. During the last days of food restriction, we buried sugar pellets in the home cage to familiarize the mice with this reward until they regularly consumed the pellets.

Next, we did a single session of shaping in which we placed the mice in a clean cage with a single scent-free well filled with cage bedding. The well had five sugar pellets on its surface and three buried in the bedding. The mice were given up to 5 min to consume all pellets. In successive trials, the pellets were buried deeper in the bedding, and the trials were repeated until the mice dug consistently for the pellet (approximately five trials).

#### Context habituation

We habituated the mice to each context for 10 min. In the experiments not involving ensemble tagging, mice were habituated to both contexts on the same day (5 min apart). In the experiments involving context tagging, mice were removed from the DOX diet and habituated first to the “tagged” context. After this exposure, they were immediately placed back on the DOX diet and, 24 hours later, habituated to the “non-tagged” context. “Tagged” and “non-tagged” contexts were counterbalanced across mice.

### Training

The task was a modified version of that used in previous studies (*17*–*19*) and had three phases: blocked context sessions (phase 1), interleaved context sessions (phase 2), and probe tests (phase 3). The blocked context sessions took place on days 1 to 4. On each day, they received 14 to 16 trials in one of the contexts, with the contexts (black versus white) switching across days. On each trial, mice were placed in the context and, after a pretrial period of 10 to 20 s, the barrier was removed allowing access to the two odor wells. One of the wells had two sugar pellets (reward) buried within the bedding. In each context, only one odor was rewarded [e.g., carvone(−) rewarded in the white context and peppermint rewarded in the black context]. Mice were given up to 3 min to find the pellets by digging in the well with the rewarded odor. The digging, our behavioral response of interest, comprised moving the bedding with the nose and paws. A correct response was scored if the animal dug in the rewarded odor well first and found the food. Otherwise, an incorrect response was scored and the subject had until the end of the trial to exhibit a correct response. The position of the wells was counterbalanced (left or right) across trials, such that no well would stay in the same position for more than two trials. The sequence for well positions was different for each session.

During days 5 to 10, mice were given interleaved context sessions. The procedures were the same as above, except that trials in the two contexts were interleaved within each day. Context order along trials was pseudo-randomized such that no more than two consecutive trials occurred in the same context. The sequence of contexts was different for each session.

### Probes

After training, mice were given a probe test or series of tests, with the number of probe tests varying by experiment. These tests occurred either in one or both training contexts and/or the novel context (table S74). In each test, we recorded the time spent digging in each well as our index for context-odor pair association memory. The behavior was scored by an experimenter unaware of experimental conditions. In chemogenetic experiments, mice were treated with C21 or VEH 60 min before testing. In optogenetic experiments, photostimulation began at the start of the pretrial period. When testing occurred in the novel and non-tagged contexts (e.g., Figs. 2, D to G, and 3, F and G, and fig. S3, D to G) test order was not counterbalanced. While this might introduce a test order confound, we did not observe any evidence for extinction at the behavioral or neural levels from the novel to the non-tagged contexts (e.g., Figs. 4, G and H, and 5, D and E).

### Perfusion, immunolabeling, and viral expression

Following the behavioral experiments, we perfused the mice transcardially with 0.1 M NaPBS (PBS) and 4% paraformaldehyde (PFA) in 0.1 M PBS (pH 7.4). The brains were extracted, postfixed in PFA overnight, and stored in PBS with 0.3% sodium azide. Brains were sliced coronally (40 μm) with a cryostat (Leica, Wetzlar, Germany).

To visualize ChR2-GFP, hM4Di-, or hM3Dq-mCherry expression, we incubated the free-floating sections in a primary antibody solution containing chicken anti-GFP (1:1000; polyclonal, GFP-1020, AVES, Tigard, OR) or rabbit anti-RFP antibody (1:1000; polyclonal, 600-401-P16, Rockland, Pottstown, PA), 0.3% Triton X-100, and 5% normal goat serum in PBS for 48 hours at 4°C, and then in a secondary antibody solution containing goat anti-chicken or anti-rabbit antibody conjugated with Alexa Fluor (1:750; Alexa Fluor 488 or 568, Thermo Fisher Scientific, Waltham, MA) and 0.3% Triton X-100 in PBS for 24 hours at 4°C to reveal and amplify the signal. Next, we counterstained the sections with 4′,6-diamidino-2-phenylindole (1:10,000; VECTASHIELD, Vector Labs, Burlingame, CA) diluted in PBS for 10 min at room temperature. Sections were washed and mounted onto gel-coated glass slides and coverslipped using PermaFluor mounting medium (VECTASHIELD, Vector Labs, Burlingame, CA). GFP or mCherry expression was verified using a confocal microscope (LSM 710; Zeiss, Oberkochen, Germany). In the majority of cases, viral expression was restricted to the DG, including both blades. In rare cases, viral expression was observed outside of the DG (e.g., in the overlaying CA1). In these cases, mice were omitted from subsequent analyses.

### Calcium imaging data processing and analysis

#### Behavioral analysis

Video of behavior was acquired at 20 Hz (20 fps). A DeepLabCut model was trained (*65*) to track the mouse head and the base of the tail, as well as the wall divider and each reward well. Coordinate positions were downsampled to 5 Hz to match the sample rate of miniature fluorescence microscope data. We defined a mouse as being in the reward zone when its head entered a 40 × 40 pixel box around each reward well.

#### Acquisition and preprocessing

Calcium images were recorded using a custom-built miniature fluorescence microscope (CHEndoscope) as previously described (*30*). Images were acquired at 20 Hz and downsampled to 5 Hz for analysis. Calcium traces were extracted from the downsampled images using CNMF-E (constrained non-negative matrix factorization for micro-endoscopic data) (*66*). Using the spatial footprints from CNMF-E, cells were registered across days using CellReg (Fig. 3D) (*67*).

#### Basic analyses

To quantify the transient rate we applied OASIS spike deconvolution to each denoised fluorescence trace within each session (*68*). We thresholded the inferred transients at a value of 0 to obtain a binarized time course of transients for each cell. The fraction of time in each session where a transient was detected was used to calculate the transient rate. Neuronal overlap between session *X* and session *Y* was calculated using the formula

$$\text{overlap}\left(X,Y\right)=\frac{\sum_{i}n_{i}\in X⋀n_{i}\in Y}{\sum_{i}n_{i}\in Y}$$

For fig. S6A, session *X* (reference) is training day 10.

### Dimensionality reduction

To visualize the high-dimensional trajectories of population activity over the course of single trials and over many training days, we used spectral embedding to reduce the dimensionality of population activity to two dimensions. Dimensionality reduction was conducted for each training day separately, and one example mouse is shown in Fig. 3H. We first downsampled population activity in time by averaging to yield a series of PVs spanning 1-s time bins. We then conducted spectral embedding using the scikit-learn implementation (*69*). Briefly, an undirected graph A was constructed, where each node was a point in time and a vertex between two nodes is present if the second node is one of the closest n neighbors to the first node in the original high-dimensional space, where *n* is the number of time bins divided by 10. Then, the Laplacian matrix *L* is calculated as *L* = *D* − *A*, where *D* is the diagonal matrix with the degree of each node in its diagonal entries, and subsequently eigen-decomposed. The eigenvectors with the lowest nonzero eigenvalues were used to project the original PVs to a two-dimensional space. Since each trial can have a variable length, we warped the reduced time courses by linear interpolation to take 1000 evenly spaced samples from the beginning of the trial to the end (reward consumption). Last, we plotted the trajectory of each trial as a curve in the reduced space and colored it according to the context in which the trial occurred.

### Within- and between-context population vector distance across training

To quantify the separation of context representation in neural space, we calculated the ratio of average pairwise cosine distances between PVs both between and within contexts over training:

$$D_{\text{between}}/D_{\text{within}}=\frac{\left(N_{c1}^{2}-N_{c1}+N_{c2}^{2}-N_{c2}\right)\sum_{c_{i}\neq c_{j}}d\left(a_{i},a_{j}\right)}{2N_{c1}N_{c2}\sum_{i\neq j,C_{i}=C_{j}}d\left(a_{i},a_{j}\right)}$$

where *c_i_* denotes the context identity (ID) for PV *a_i_*, and *N*_*c*1_ and *N*_*c*2_ denote the number of PVs in context 1 and context 2, respectively, where

$$d\left(a_{i},a_{j}\right)=1-\frac{a_{i}∙a_{j}}{∥a_{i}∥∥a_{j}∥}$$

For a given mouse and training day, we calculated the trial-averaged fluorescence for each cell yielding one PV per trial. We excluded any time bins corresponding to when the mouse was in a reward zone when calculating this average. We then used the above formula to calculate a PV distance for each mouse and training day. Last, the chance level was calculated for each mouse and training day by repeating the above procedure 10 times with randomly permuted context IDs, and the resulting PV distances were averaged across permutations.

### Latent space probability distribution distance across training

To quantify the divergence of context representations in a latent space across training, for each training day and each mouse, we computed the PC analysis (PCA) on 200-ms time bins across all trials on that training day. We then projected each time bin to an *N*-dimensional space using the first five PCs and *z*-scored the resulting PC scores along each dimension. For each context, we then estimated a nonparametric, *N*-dimensional probability distribution using kernel density estimation with a Gaussian kernel. The kernel size was determined for each mouse using cross-validation (CV). For each training day, a random 10% subsample of the time bins was selected for the CV procedure. We then tested a range of kernel sizes in a fourfold CV procedure and calculated which kernel size maximized the likelihood of the held-out data. The final kernel size for a given mouse was chosen by averaging the optimal kernel size across all training days. Once the probability distribution for each context was estimated, we calculated a distribution distance using a symmetric version of the Kullback-Leibler divergence

$$\begin{array}{cc} D\left(P\left(p|C_{1}\right),P\left(p|C_{2}\right)\right) & =\sum_{p\in X}P\left(p|C_{1}\right)\text{log}\left(\frac{P\left(p|C_{1}\right)}{P\left(p|C_{2}\right)}\right)\\ & +\sum_{p\in X}P\left(p|C_{2}\right)\text{log}\left(\frac{P\left(p|C_{2}\right)}{P\left(p|C_{1}\right)}\right) \end{array}$$

where *p* is the *N*-dimensional PV embedded in PC space, and *C*_1_ and *C*_2_ are the tagged and non-tagged contexts, respectively. *X* is the set of sampled points in the *N*-dimensional probability distribution domain, which was chosen to be evenly spaced points between −10 and 10 in each dimension, with the spacing chosen to be the cross-validated kernel size calculated earlier. We excluded any time bins corresponding to when the mouse was in a reward zone when performing this calculation.

### Population vector analysis

To calculate the PVs of average activity, we separated time points by context and integrated the denoised calcium traces from CNMF-E across time, yielding one average activity vector for each context on each training day, and one vector for each condition (VEH, C21) on each probe day. For all training day sessions, we excluded time bins where the animal was within the reward zone to minimize the influence of scent representations in our analysis. In addition, we excluded time points in the probe session when the mouse was digging or sniffing to minimize the influence of behavioral performance on our analyses. To calculate the similarity between two average activity PVs, we first restricted the vector to cells that were observed in both sessions. For each cell present in both sessions, we calculated its *z*-scored activity

$$z_{i}=\frac{\left(a_{i}-\bar{a}\right)}{\sigma \left(a\right)}$$

where *a_i_* is the integrated calcium trace for the *i*th neuron, $\bar{a}$ is the average integrated calcium trace for all cells in that session, and σ(*a*) is the SD of integrated calcium traces in that session. The single-cell PV correlation between sessions 1 and 2 is then

$$c_{i}=z_{i,1}z_{i,2}$$

### Normalized correlation analysis

To calculate the normalized correlation matrix (NCM) for a particular session, we first calculated the Pearson correlation coefficient for each pair of neurons. For all training day sessions, we excluded time bins where the animal was within the reward zone. To dissociate the effects of, and control for, differences in average activity or transient shape, we normalized each pair’s correlation coefficient to a null distribution created by circularly shuffling each calcium trace. Similar to the PV analysis, we calculated single-cell correlation coefficients between two sessions of interest by first restricting the analysis to cells detected in both sessions. For each cell, we then have a vector containing its normalized correlations to every other cell detected in both sessions. To get a single-cell correlation, we then calculated the Pearson correlation coefficient between these two vectors from the same cell in each session of interest.

### Ensemble analysis

To extract ensembles from a particular session, we used a PCA-based method. For all training day sessions, we excluded time bins where the mouse was within the reward zone. We eigen-decomposed the Pearson correlation matrix from the population activity in a particular session. We subsequently created a null distribution of eigenvalues by circularly shuffling calcium traces and eigen-decomposing their correlation matrices. We considered an eigenvector a significant ensemble if its corresponding eigenvalue exceeded one SD from the mean of the null distribution of eigenvalues. Once we extracted ensembles from a particular session, we downsampled a session of interest into 1-s time bins. For each 1-s activity vector at time *t* and the *i*th ensemble, we calculated the dot product between the ensemble eigenvector and the activity vector

$$e_{i,t}=a_{t}∙e_{i}$$

where **a***_t_* is the population activity vector in the session of interest in time bin *t*, and **e***_i_* is the ensemble PV of the *i*th ensemble detected in the reference session.

We then calculated an ensemble activation score by normalizing the dot product similarity to the mean and SD of a null distribution of dot products created by 1000 cell ID shuffles of the ensemble vector. We considered an ensemble to be significantly active at time bin t if the ensemble activation score was greater than 2 (i.e., the actual dot produced exceeded two null distribution SDs away from the null distribution mean).

### Synchronous calcium events

To detect synchronous calcium events (SCEs) for each session, we calculated the fraction of active cells in each time bin (*40*, *41*). We then filtered this trace with a Gaussian filter width kernel sigma of 50 ms. We then *z*-scored this value, and whichever time bins exceeded a *z*-score of 2 were considered significant SCEs. To estimate the temporal relationship between significant ensemble activations and SCEs, we calculated the correlation between the temporal trace of SCEs and ensemble activation (for each ensemble) after smoothing with a Gaussian filter with a kernel sigma of 1 s. We compared this correlation value to the average correlation of 50 iterations of circular shuffles of the ensemble activation traces.

## Supplementary Materials

### This PDF file includes:

Supplementary Text Tables S1 to S74 Figs. S1 to S6 References
