# Supplementary material for: Dentate gyrus ensembles gate context-dependent neural states and memory retrieval
Source: Sci Adv. 2024 Aug 2;10(31):eadn9815. doi: 10.1126/sciadv.adn9815 (PMC11296340; doi:10.1126/sciadv.adn9815)
Supplement: Supplementary file 1 — Supplementary Text Tables S1 to S74 Figs. S1 to S6 References [file sciadv.adn9815_sm.pdf]

Science Advances
AAAS

Supplementary Materials for

Dentate gyrus ensembles gate context-dependent neural states and memory retrieval

Cesar A. O. Coelho *et al.*

Corresponding author: Sheena A. Josselyn, sheena.josselyn@sickkids.ca;
Paul W. Frankland, paul.frankland@sickkids.ca

Sci. Adv. **10**, eadn9815 (2024)
DOI: 10.1126/sciadv.adn9815

This PDF file includes:

Supplementary Text
Tables S1 to S74
Figs. S1 to S6
References

SUPPLEMENTARY MATERIALS

SUPPLEMENTARY TEXT

Statistical report

Statistical analysis for behavioral data

In the experiment involving the contextual fear conditioning (CFC) task, we used the percentage of time freezing as our behavioral measure during both training and test. We analyzed the training data using a linear regression model testing the effects of treatment group (GFP or ChR2) and phase (baseline, shock, post-shock) on percent time freezing. We calculated family wise effects in the model using an ANOVA, and used estimated marginal means (EMMEANS) to assess the contrasts of interest. The p values of the EMMEANS were corrected using a false discovery rate (fdr) correction. We analyzed the test data in the same manner, but tested the effects of group (GFP or ChR2) and light stimulation (OFF or ON) in the linear model.

In the experiments involving the context-odor pair associate task, we used the trial response (correct or wrong) as behavioral measure during the training phase. We analyzed the training data using generalized linear mixed models (GLMM) with binomial distribution family and logit link. The model was fit by a Laplacian approximation of maximum likelihood. Our analysis relied on a nested structure with trial level within the subject level (mouse). We evaluated the fixed effects of day and context on the likelihood of correct response, with random effects at the subject level. When part of the experimental design, we also included the fixed effect of group. We built models both with and without the interaction terms and chose the final model fit based on the Akaike information criterion (AIC), however, the model fit without interaction terms presented the best AIC values in all experiments.

We analyzed the probe tests using the percent time digging as our main behavioral measure. We used a GLMM fit by restricted maximum likelihood (REML), and t-tests with Satterthwaite method for confidence intervals. As in the training data, this model relied on a nested structure with trials grouped at the subject level (mouse). We evaluated the effects of group, stimulation (OFF or ON for optogenetic experiments, VEH or C21 for chemogenetic experiments) and odor (RE, rewarded, or NON, non-rewarded) as predictors of percent time digging, with random effects grouped at the subject level. We used EMMEANS to test the contrasts of interest as described above.

During the probes, we also analyzed the discrimination index, defined as the difference of percent time spent digging in each well divided by their sum [(RE - NON)/(RE + NON)]. We used the GLMM as above, but tested the effects of group and stimulation (OFF or ON; VEH or C21) as predictors of discrimination. We used EMMEANS as described above to test the conditions against chance levels (zero) and then to compare the contrasts of interest.

The statistical analysis was performed using R 4.0.4 (R Core Team, 2021) and the packages: dplyr (*70*), lme4 (*71*), lmerTest (*72*), emmeans (*73*), ggplot2 (*74*) and ggpubr (*75*).

Experimental design and statistical report

Experiment 1: Behavioral profile in the context-odor pair associate task

We first characterized the general behavioral profile observed during the training and probe phases of the context-odor pair associate task (*N* = 8; Fig. 1, A to D). Mice were food restricted to 90% free-feed body weight, shaped to dig for food pellets, trained and memory probed. Memory was probed in both contexts (order and exposure counterbalanced).

In the training data, the GLMM evaluating the effect of day and context on the likelihood of correct response was significant ($\chi 2(2, N = 1232) = 107.33$, $p < 0.0001$, R2 = 0.31, nagelkerke-R2 = 0.33; table S1). The likelihood of correct response was not affected by context, but significantly increased by a ratio of 1.38 per day, accounting for learning the context-odor pair associate task over the training days (Fig. 1, A and B).

Table S1: GLMM testing context, day as predictors of (correct) response during training

	**response**				
Predictors	*Odds Ratios*	*std. Error*	*CI*	*z value*	*p value*
(Intercept)	1.63 *	0.35	1.08 – 2.47	2.30	**0.021**
context [W]	1.04	0.20	0.72 – 1.51	0.22	0.828
day	1.38 ***	0.05	1.28 – 1.48	8.72	**<0.001**
**Random Effects**					
σ^2	3.29				
$\tau_{00\ mouse}$	0.06				
ICC	0.02				
N_{mouse}	8				
Observations	1232				
Marginal R^2 / Conditional R^2	0.314 / 0.327				
Deviance	763.515				
AIC	771.515				
log-Likelihood	-381.757				

** p<0.05   ** p<0.01   *** p<0.001*

In the probe, a GLMM testing whether odor predicted percent time digging explained significantly more variance than the random effects ($\chi 2(1, N = 80) = 4.28$, $p = 0.0386$, R2 = 0.05, nagelkerke-R2 = 0.10; table S2), and showed evidence that the mice dug for longer in the rewarded well than in the non-rewarded well. In the discrimination analysis, the EMMEANS revealed a discrimination index statistically above chance level (emmean = 0.18, std. error = 0.058, df = 7, 95%CI = 0.04 - 0.31, t.ratio = 3.06, p = 0.0182). These results indicate that digging behavior and discrimination scores are measures of context-specific memory in the context-odor pair associate task (Fig. 1, C and D).

Table S2: GLMM evaluating the effect of context on percent time dug during probes

	time_dug				
Predictors	*Estimates*	*std. Error*	*CI*	*z value*	*p value*
(Intercept)	6.64 ***	0.95	4.74 – 8.53	6.96	**<0.001**
odor [RE]	2.46 *	1.18	0.11 – 4.81	2.08	**0.040**
**Random Effects**					
σ^2	27.82				
$\tau_{00\ mouse}$	1.71				
ICC	0.06				
N_{mouse}	8				
Observations	80				
Marginal R^2 / Conditional R^2	0.049 / 0.104				
Deviance	494.889				
AIC	499.486				
log-Likelihood	-245.743				

** p<0.05 ** p<0.01 *** p<0.001*

Experiment 2: Chemogenetic inhibition of 'context only' ensembles inhibits context-specific behavior in the tagged context

We sought to test whether dentate gyrus ensembles encoding the tagged context are required to recall the appropriate context-odor pair associate to guide digging behavior. Rather than using optogenetics, we used chemogenetics to interrogate dentate gyrus context ensembles (Fig. 1, E to H). We microinjected RAM-hM4D viral vector in mice (*N* = 8) under DOX diet (200 mg/kg). After recovery, mice were food restricted and shaped (DOX on). Mice were removed from DOX diet for 48 h, and one of the contexts was tagged during a habituation session. Mice were returned to the DOX diet and, 48 h later, habituated to the other context. Mice were then trained on the context-odor pair associate task for 10 days. On days 11-12, we tested mice in the tagged context 1 h after an injection of C21 or VEH (Fig. 1E).

In the training data, the GLMM indicated that the effect of day and context on the likelihood of correct response was significant (Χ2(2, N = 1120) = 180.55, p < 0.0001, R2 = 0.26, nagelkerke-R2 = 0.26; table S3). The likelihood of correct response was not significantly affected by context, but significantly increased by a ratio of 1.45 per day, showing evidence of context-odor paired associates learning across the days (Fig. 1F).

Table S3: GLMM evaluating the effect of context and day on the response behavior during training

	response				
Predictors	*Odds Ratios*	*std. Error*	*CI*	*z value*	*p value*
(Intercept)	0.59 **	0.10	0.43 – 0.81	-3.25	**0.001**
context [W]	0.83	0.13	0.62 – 1.13	-1.18	0.239
day	1.45 ***	0.05	1.36 – 1.54	11.87	**<0.001**
**Random Effects**					
σ^2	3.29				
$\tau_{00\ mouse}$	0.01				
ICC	0.00				
N_{mouse}	8				
Observations	1120				
Marginal R^2 / Conditional R^2	0.256 / 0.259				
Deviance	1056.626				
AIC	1064.626				
log-Likelihood	-528.313				

* *p<0.05* ** *p<0.01* *** *p<0.001*

In the tagged context probe test, a GLMM testing whether stimulation (VEH, C21) and odor (rewarded, non-rewarded) predicted the percent time digging was significant (Χ2(3, N = 160) = 49.57, p > 0.0001, R2 = 0.24, nagelkerke-R2 = 0.34; Table SR3.2). Using the EMMEANS analysis to test the contrasts of interest (table S4 and Fig. 1G), we observed that, after VEH, mice dug longer in the rewarded well compared to the non-rewarded well, but dug equivalently in both wells after C21 injection. We also observed that the time dug in the rewarded well was longer after VEH than after C21 injection, whereas mice dug for longer in the non-rewarded well after C21 injection compared to VEH injection. These results provide statistical evidence that dentate gyrus ensembles guide context-specific behavior.

Table S4: GLMM evaluating the effect of stimulation and odor on percent time dug during tagged context probes

	time_dug				
Predictors	*Estimates*	*std. Error*	*CI*	*z value*	*p value*
(Intercept)	6.87 **	2.31	2.30 – 11.44	2.97	**0.003**
stimulation [C21]	11.47 ***	2.46	6.61 – 16.32	4.67	**<0.001**
odor [RE]	18.09 ***	2.46	13.24 – 22.94	7.37	**<0.001**
stimulation [C21] × odor [RE]	-22.46 ***	3.47	-29.32 – -15.60	-6.47	**<0.001**
**Random Effects**					
σ^2	120.61				
$\tau_{00\ mouse}$	18.69				
ICC	0.13				
N_{mouse}	8				
Observations	160				
Marginal R^2 / Conditional R^2	0.238 / 0.341				
Deviance	1228.100				
AIC	1226.974				
log-Likelihood	-607.487				

* *p<0.05   ** p<0.01   *** p<0.001*

Table S5: EMMEANS of contrasts of interest during tagged context probe

Odor	*Stim*	*Contrast*	*Estimate*	*SE*	*df*	*lower.CI*	*upper.CI*	*t ratio*	*p value*
NON	.	VEH - C21	-11.47	2.46	149	-17.67	-5.26	-4.67	<0.0001
RE	.	VEH - C21	10.99	2.46	149	4.78	17.20	4.48	0.0001
.	VEH	NON - RE	-18.09	2.46	149	-24.30	-11.88	-7.37	<0.0001
.	C21	NON - RE	4.37	2.46	149	-1.84	10.58	1.78	0.3093

The discrimination analysis supported the percent time digging analysis. A GLMM testing the effect of stimulation (C21 or VEH) on discrimination index was significant ($\chi2(1, N = 80) = 38.47$, $p < 0.0001$, R2 = 0.36, nagelkerke-R2 = 0.43). The EMMEANS showed a discrimination significantly above chance levels after VEH injection, but not after C21 (table S5 and Fig. 1H). Contrasting stimulation conditions, discrimination was significantly higher after VEH injection than after C21 (emmean = 0.72, std. error = 0.10, df = 71, 95% CI = 0.52 - 0.92, t.ratio = 7.08, $p < 0.0001$). Together, these results indicate that dentate gyrus ensembles are necessary for context-specific discriminative memory.

Table S6: EMMEANS comparing discrimination to chance (zero) in the stimulation conditions during tagged context probes

stimulation	*emmean*	*SE*	*df*	*lower.CL*	*upper.CL*	*t.ratio*	*p value*
VEH	0.58	0.09	14	0.35	0.81	6.31	<0.0001
C21	-0.14	0.09	14	-0.37	0.09	-1.49	0.1578

Experiment 3: Chemogenetic stimulation of 'context only' ensembles drives context-specific behavior in a novel context and the non-tagged context

This experiment used a chemogenetic approach to address whether stimulation of 'context only' ensembles drives context-specific behavior in a novel context and the non-tagged context (*N* = 8; Fig. 2, A to G).

In the training data, the GLMM testing the effect of day and context in the likelihood of (correct) response was significant model ($\chi^2(2, N = 1232) = 196.94$, $p < 0.0001$, $R^2 = 0.33$, nagelkerke-$R^2 = 0.36$; table S7). The likelihood of correct response was not significantly affected by context, but significantly increased by a ratio of 1.48 per day, showing evidence of learning the context-odor pair associates over the days (Fig. 2C).

Table S7: GLMM evaluating the effect of context and day on the response behavior during context-odor pair associate training

	response				
Predictors	*Odds Ratios*	*std. Error*	*CI*	*z value*	*p value*
(Intercept)	0.80	0.17	0.53 – 1.21	-1.07	0.286
context [W]	0.80	0.13	0.57 – 1.10	-1.37	0.172
day	1.48 ***	0.05	1.39 – 1.58	11.75	**<0.001**
**Random Effects**					
σ^2	3.29				
$\tau_{00\ mouse}$	0.14				
ICC	0.04				
N_{mouse}	8				
Observations	1232				
Marginal R^2 / Conditional R^2	0.330 / 0.357				
Deviance	945.562				
AIC	953.562				
log-Likelihood	-472.781				

* *p<0.05* ** *p<0.01* *** *p<0.001*

In the novel context probe, we tested whether stimulation and odor predicted percent time digging. Our overall model explained significantly more variance than the random effects ($\chi^2(3, N = 160) = 16.74$, $p = 0.0008$, $R^2 = 0.22$, nagelkerke-$R^2 = 0.27$; summarized in table S8). We tested the contrasts of interest via EMMEANS (table S9) and observed that mice dug longer in the rewarded well compared to the non-rewarded after C21, but not after VEH injection. Mice also dug longer in the rewarded odor-well after C21 than after VEH injection, but dug equivalently in the non-rewarded well (Fig. 2D).

Table S8: GLMM testing stimulation and odor as predictors of percent time dug during novel context probes

	time_dug				
Predictors	*Estimates*	*std. Error*	*CI*	*z value*	*p value*
(Intercept)	9.62 ***	2.22	5.24 – 14.00	4.34	**<0.001**
stimulation [C21]	-1.19	2.06	-5.26 – 2.88	-0.58	0.565
odor [RE]	-1.20	2.06	-5.27 – 2.87	-0.58	0.561
stimulation [C21] × odor [RE]	8.51 **	2.91	2.75 – 14.26	2.92	**0.004**
**Random Effects**					
σ^2	84.91				
$\tau_{00\ mouse}$	22.40				
ICC	0.21				
N_{mouse}	8				
Observations	160				
Marginal R^2 / Conditional R^2	0.079 / 0.272				
Deviance	1175.355				
AIC	1175.207				
log-Likelihood	-581.604				

* $p<0.05$ ** $p<0.01$ *** $p<0.001$

Table S9: EMMEANS of contrasts of interest during novel context probe

Odor	*Stim*	*Contrast*	*Estimate*	*SE*	*df*	*lower.CI*	*upper.CI*	*t ratio*	*p value*
NON	.	*VEH - C21*	1.19	2.06	149	-4.02	6.40	0.58	0.5653
RE	.	*VEH - C21*	-7.32	2.06	149	-12.53	-2.11	-3.55	0.0010
.	*VEH*	*NON - RE*	1.20	2.06	149	-4.01	6.41	0.58	0.5653
.	*C21*	*NON - RE*	-7.31	2.06	149	-12.52	-2.10	-3.55	0.0010

The discrimination analysis of the novel context probes supported the percent time digging analysis. A GLMM testing the effect of stimulation (VEH or C21) on discrimination index was significant (Χ2(2, N = 80) = 7.23, p = 0.0269, R2 = 0.06, nagelkerke-R2 = 0.10). The EMMEANS showed a discrimination at chance level (zero) after VEH injections and above chance level after C21 injections (table S10 and Fig. 2E). There was also a significantly higher discrimination after C21 injection, contrasting stimulation conditions (emmean = 0.24, std. error = 0.11, df = 71, 95% CI = 0.02 - 0.46, t.ratio = 2.19, p = 0.0317). These results replicate our findings in Experiment 5, and provide evidence, using chemogenetic methods, that stimulation of context-only ensembles in the dentate gyrus can guide context-specific behavior in a novel context.

Table S10: EMMEANS comparing discrimination to chance (zero) in the stimulation conditions during novel context probes

stimulation	*emmean*	*SE*	*df*	*lower.CL*	*upper.CL*	*t.ratio*	*p value*

VEH	-0.03	0.08	25	-0.22	0.15	-0.41	0.6840
C21	0.21	0.08	25	0.02	0.40	2.69	0.0250

In the non-tagged context probe, a GLMM testing whether stimulation and odor predicted percent time digging explained significantly more variance than the random effects (χ2(3, N = 160) = 17.26, p = 0.0006, R2 = 0.09, nagelkerke-R2 = 0.23; table S11). The EMMEANs revealed a double dissociation in the stimulation conditions. Mice dug significantly longer in the non-rewarded (compared to non-rewarded) well after VEH injection, and this pattern was reversed following the C21 injection (Table S12 and Fig. 2F). Note that the non-rewarded well here refers to the well that was rewarded in the non-tagged context, and it was expected that mice dug longer in that well after control injection (VEH).

Table S11: GLMM evaluating the effect of stimulation and odor on percent time dug during the non-tagged context probes

	time_dug				
Predictors	*Estimates*	*std. Error*	*CI*	*z value*	*p value*
(Intercept)	24.38 ***	4.23	16.03 – 32.73	5.77	**<0.001**
stimulation [C21]	-12.14 **	4.27	-20.58 – -3.70	-2.84	**0.005**
odor [RE]	-10.46 *	4.27	-18.90 – -2.02	-2.45	**0.015**
stimulation [C21] × odor [RE]	25.20 ***	6.04	13.26 – 37.13	4.17	**<0.001**
**Random Effects**					
σ^2	364.95				
τ_{00} mouse	70.01				
ICC	0.16				
N mouse	8				
Observations	160				
Marginal R^2 / Conditional R^2	0.086 / 0.233				
Deviance	1406.582				
AIC	1400.862				
log-Likelihood	-694.431				

* *p<0.05* ** *p<0.01* *** *p<0.001*

Table S12: EMMEANS of contrasts of interest during the non-tagged context probes

Odor	*Stim*	*Contrast*	*Estimate*	*SE*	*df*	*lower.CI*	*upper.CI*	*t ratio*	*p value*
NON	.	VEH - C21	12.14	4.27	149	1.34	22.94	2.84	0.0068
RE	.	VEH - C21	-13.05	4.27	149	-23.85	-2.25	-3.06	0.0053
.	VEH	NON - RE	10.46	4.27	149	-0.34	21.26	2.45	0.0155
.	C21	NON - RE	-14.73	4.27	149	-25.53	-3.93	-3.45	0.0029

The discrimination analysis of the non-tagged context probes supported the percent time digging analysis. A GLMM testing the effect of stimulation (VEH or C21) on discrimination index was significant ($\chi^2(2, N = 80) = 13.48$, $p = 0.0012$, $R^2 = 0.15$, nagelkerke-$R^2 = 0.15$). The EMMEANS revealed a discrimination significantly below chance level (zero) after VEH injection that shifted to significantly above chance level after C21 injections (table S13 and Fig. 2G). Contrasting the conditions, discrimination after C21 injection was significantly higher than after VEH (emmean = 0.48, std. error = 0.13, df = 71, 95% CI = 0.22 - 0.73, t.ratio = 3.73, $p = 0.0004$). These results provide evidence that chemogenetic interrogations of context-encoding dentate gyrus cells regulate context-specific behaviors and disambiguate similar circumstances, replicating the optogenetics findings from Experiment 5.

Table S13: EMMEANS comparing discrimination to chance (zero) in the stimulation conditions during non-tagged context probes

stimulation	*emmean*	*SE*	*df*	*lower.CL*	*upper.CL*	*t.ratio*	*p value*
VEH	-0.20	0.09	25	-0.41	0.02	-2.20	0.0371
C21	0.28	0.09	25	0.06	0.49	3.08	0.0099

Experiment 4: Stimulation of context fear ensembles drives freezing behavior in a novel context

In this experiment, we examined the dose of DOX to use in food restricted mice to allow specific expression using the RAM virus. We tested whether the tagging specificity of the RAM virus is maintained under the food restriction procedure used in the context-odor pair associate task, and whether we could replicate, under these conditions, previous findings on photo-stimulation of contextual fear encoding ensembles. These experiments established that using concentration of 200 mg/kg of DOX in the diet led to effective tagging in food-restricted mice (fig. S1).

Next, we microinjected mice with AAV-RAM-CHR2-eYFP ($N = 8$) or control AAV-RAM-GFP ($N = 6$) under DOX (200 mg/kg). Following recovery mice were put at 90% free-feed body weight (on DOX). Mice were removed from DOX diet for 48 h and were given contextual fear training to “tag” the experience‘. Mice were put back on DOX diet after training. After 48 h, we photo-stimulated the tagged neuronal ensembles in a novel context (fig. S2A).

During the training, we obtained a significant linear model ($F(5, 36) = 11.92$, $p < 0.0001$, $R^2 = 0.62$, Adjusted-$R^2 = 0.57$) to which the ANOVA showed a significant effect of phase ($F(2, 24) = 64.87$, $p < 0.0001$), but no effect of group ($F(1, 12) = 0.03$, $p = 0.8810$) or group x phase interaction ($F(2, 24) = 2.42$, $p = 0.1120$), indicating an increase in freezing across the phases (higher freezing after the shock) (table S14) that was equivalent between the two groups (fig. S2B). These findings suggest both groups acquired the context fear conditioning equivalently.

Table S14: EMMEANS comparing phase conditions during CFC training

contrast	*estimate*	*SE*	*df*	*lower.CL*	*upper.CL*	*t.ratio*	*p.value*
baseline - shock	-17.60	6.86	36	-34.83	-0.37	-2.57	0.0439
baseline - post	-51.31	6.86	36	-68.54	-34.08	-7.48	<0.0001
shock - post	-33.71	6.86	36	-50.94	-16.48	-4.91	0.0001

During the test in a novel context, we obtained a significant linear model ($F(3, 52) = 31.86$, $p < 0.0001$, $R2 = 0.65$, Adjusted-$R2 = 0.63$), which the ANOVA revealed a significant effect of group ($F(1, 52) = 52.95$, $p < 0.0001$), stimulation ($F(1, 52) = 22.92$, $p < 0.0001$) and a group x stimulation interaction ($F(1, 52) = 19.71$, $p < 0.0001$). In the EMMEANS, when contrasting stimulation, the GFP group exhibited equivalent freezing during both stimulation windows, whereas the ChR2 group showed higher freezing during stimulation ON compared to OFF. Contrasting the groups, there was no significant group difference in freezing during stimulation OFF, but a significantly higher freezing in ChR2 group during stimulation ON (table S15 and fig. S2C). These findings indicate that photo-stimulating the ensemble tagged during context fear conditioning increased freezing behavior, replicating the previous findings (*21*) but using mice that were food restricted.

Table S15: EMMEANS comparing group and stimulation conditions during CFC test

group	stim	contrast	estimate	SE	df	lower.CL	upper.CL	t.ratio	p.value
GFP	.	OFF - ON	1.11	5.01	52	-11.86	14.08	0.22	0.8255
ChR2	.	OFF - ON	-28.33	4.34	52	-39.57	-17.10	-6.53	<0.0001
.	OFF	GFP - ChR2	-9.41	4.69	52	-21.55	2.73	-2.01	0.1001
.	ON	GFP - ChR2	-38.85	4.69	52	-50.99	-26.72	-8.28	<0.0001

Experiment 5: Photo-stimulation of 'context only' ensembles drives context-specific behavior in a novel context and in the non-tagged context

In the previous experiment, we tagged dentate gyrus ensembles active during a single-context training session. But one might argue that the behavior driven by the photostimulation of session-encoding ensembles may have been driven by elements common to both contexts, such as the odors, wells, rewards, etc. In this experiment, we tested whether photostimulation of 'context-only' ensembles could drive context-specific behavior both in a novel context and in

the non-tagged context. To this end, we tagged the context during habituation, before any odor-context association learning had taken place (fig. S3). Note that there are two hypotheses in this design. First, photostimulation of context-encoding ensembles can drive context-specific memory. Second, ensembles encoding the context-odor pair associates still rely on populations present in the initial encoding (those tagged).

Mice were microinjected with virus expressing RAM-ChR2 (*N* = 8) or RAM-GFP (*N* = 8, fig. S3A). After recovery and food restriction to 90% free-feed body weight, mice were removed from the DOX diet for 48 h, placed in one context and then replaced on the DOX diet. Mice were habituated to the other context 48 h later. Mice were then trained as previously. Mice were tested in two probe sessions in a novel context, one with and one without photostimulation (counterbalanced). On the following day, we ran an intermixed context training session to mitigate possible effects of these probes sessions (e.g., extinction). Lastly, we tested mice in two non-tagged probe sessions, one with and one without photostimulation (fig. S3B).

In the training data, the GLMM performed to evaluate the effect of day, context and group on the likelihood of correct response explained significantly more variance than the Random models ($\chi2(3, N = 2688) = 515.18$, $p < 0.0001$, $R2 = 0.35$, nagelkerke-$R2 = 0.39$; table S16). The likelihood of correct response was not significantly affected by context or group, but significantly increased by a ratio of 1.39 per day, providing evidence for learning the context-odor pair associates (fig. S3C).

Table S16: GLMM testing context, day and group as predictors of (correct) response during training

	response				
Predictors	*Odds Ratios*	*std. Error*	*CI*	*z value*	*p value*
(Intercept)	0.44 ***	0.09	0.29 – 0.66	-4.02	**<0.001**
context [W]	1.08	0.11	0.88 – 1.32	0.74	0.462
day	1.39 ***	0.02	1.34 – 1.44	18.39	**<0.001**
group [ChR2]	1.57	0.40	0.95 – 2.59	1.76	0.079
**Random Effects**					
σ^2	3.29				
$\tau_{00\ mouse}$	0.22				
ICC	0.06				
N_{mouse}	16				
Observations	2688				
Marginal R^2 / Conditional R^2	0.351 / 0.392				
Deviance	2326.138				
AIC	2336.138				
log-Likelihood	-1163.069				

** p<0.05   ** p<0.01   *** p<0.001*

In the novel context probe, we tested whether stimulation, odor and group predicted percent time digging. The GLMM yielded a significant model ($\chi2(7, N = 320) = 51.90$, $p < 0.0001$, $R2 = 0.15$, nagelkerke-$R2 = 0.23$; summarized in table S17). We tested the contrasts of interest using EMMEANS (table S18). The mice in the GFP group dug equivalently in both wells during both

stimulation sessions, presenting no within-group differences. The mice in the ChR2 group dug longer in the rewarded well during stimulation ON compared to OFF, but equivalently in the non-rewarded well. During stimulation ON, they also dug longer in the rewarded well than in the non-rewarded one, but not during stimulation OFF. The only group difference that survived the p correction was that the GFP group dug longer in the rewarded well during stimulation OFF (fig. S3D).

Table S17: GLMM evaluating the effect of group, stimulation and odor on percent time dug during novel context probes

	time_dug				
Predictors	*Estimates*	*std. Error*	*CI*	*z value*	*p value*
(Intercept)	7.05 ***	1.67	3.76 – 10.34	4.22	**<0.001**
group [ChR2]	-4.82 *	2.36	-9.47 – -0.17	-2.04	**0.042**
stimulation [ON]	2.24	1.90	-1.50 – 5.97	1.18	0.239
odor [RE]	1.80	1.90	-1.94 – 5.53	0.95	0.344
group [ChR2] × stimulation [ON]	1.29	2.68	-4.00 – 6.57	0.48	0.632
group [ChR2] × odor [RE]	-2.14	2.68	-7.43 – 3.14	-0.80	0.425
stimulation [ON] × odor [RE]	-1.49	2.68	-6.78 – 3.79	-0.56	0.579
(group [ChR2] × stimulation [ON]) × odor [RE]	9.76 *	3.80	2.29 – 17.23	2.57	**0.011**
**Random Effects**					
σ^2	72.08				
$\tau_{00\ mouse}$	7.89				
ICC	0.10				
N_{mouse}	16				
Observations	320				
Marginal R^2 / Conditional R^2	0.147 / 0.231				
Deviance	2287.488				
AIC	2285.855				
log-Likelihood	-1132.927				

* p<0.05 ** p<0.01 *** p<0.001

Table S18: EMMEANS of contrasts of interest in percent time dug during novel context probe

Group	*Odor*	*Stim*	*Contrast*	*Estimate*	*SE*	*df*	*lower.CI*	*upper.CI*	*t ratio*	*p value*
GFP	*NON*	*.*	*ON - OFF*	2.24	1.90	298	-3.24	7.72	1.18	0.3593
GFP	*RE*	*.*	*ON - OFF*	0.75	1.90	298	-4.74	6.23	0.39	0.8340
ChR2	*NON*	*.*	*ON - OFF*	3.53	1.90	298	-1.96	9.01	1.86	0.1544
ChR2	*RE*	*.*	*ON - OFF*	11.79	1.90	298	6.31	17.28	6.21	<0.0001
GFP	*.*	*ON*	*NON - RE*	-0.31	1.90	298	-5.79	5.18	-0.16	0.8720

GFP	*.*	*OFF*	*NON - RE*	-1.80	1.90	298	-7.28	3.68	-0.95	0.4589
ChR2	*.*	*ON*	*NON - RE*	-7.93	1.90	298	-13.41	-2.44	-4.17	0.0002
ChR2	*.*	*OFF*	*NON - RE*	0.34	1.90	298	-5.14	5.83	0.18	0.8720
.	*NON*	*ON*	*GFP - ChR2*	3.53	2.36	51	-3.56	10.62	1.50	0.2418
.	*NON*	*OFF*	*GFP - ChR2*	4.82	2.36	51	-2.27	11.91	2.04	0.1397
.	*RE*	*ON*	*GFP - ChR2*	-4.09	2.36	51	-11.18	3.00	-1.73	0.1792
.	*RE*	*OFF*	*GFP - ChR2*	6.96	2.36	51	-0.13	14.05	2.95	0.0193

The discrimination analysis of the novel context probes further confirmed the percent time digging analysis. A GLMM testing the effect of group and stimulation on discrimination index was significant ($\chi 2(1, N = 142) = 14.88$, $p = 0.0001$, $R2 = 0.11$, nagelkerke-$R2 = 0.12$). The EMMEANS indicated discrimination at chance level (zero) in the GFP group during both stimulation conditions, whereas in the ChR2 group, discrimination was at chance level during stimulation OFF and increased significantly above chance levels during stimulation ON (table S19). Contrasting stimulation conditions in the ChR2 group, there was a significantly higher discrimination during stimulation ON, but no difference in the GFP group. Contrasting the groups, there was a higher discrimination in the ChR2 group during stimulation ON, but no difference during stimulation OFF (table S20 and fig. S3E). Taken together, these results provide evidence that photostimulation of (tagged) context-encoding dentate gyrus ensembles drove behavior from chance levels (as would be expected in the novel context) to what would be expected in the tagged context, suggesting that dentate gyrus ensembles can drive context-specific memory recall.

Table S19: EMMEANS comparing discrimination to chance (zero) across group and stimulation during novel context probes

stimulation	*group*	*emmean*	*SE*	*df*	*lower.CL*	*upper.CL*	*t.ratio*	*p value*
OFF	*GFP*	0.17	0.09	44	-0.08	0.42	1.80	0.1567
ON	*GFP*	-0.07	0.10	46	-0.32	0.18	-0.77	0.4481
OFF	*ChR2*	-0.14	0.11	59	-0.42	0.14	-1.32	0.2580
ON	*ChR2*	0.38	0.09	44	0.13	0.62	4.03	0.0009

Table S20: EMMEANS of contrasts of interest in discrimination during novel context probe

Group	*Stim*	*Contrast*	*Estimate*	*SE*	*df*	*lower.CI*	*upper.CI*	*t ratio*	*p value*
GFP	*.*	*OFF - ON*	0.24	0.13	127	-0.10	0.58	1.81	0.2907

ChR2	*.*	*OFF - ON*	-0.52	0.14	129	-0.89	-0.16	-3.64	0.0016
.	*OFF*	*GFP - ChR2*	0.31	0.14	52	-0.06	0.68	2.18	0.1368
.	*ON*	*GFP - ChR2*	-0.45	0.13	45	-0.80	-0.10	-3.38	0.0061

In the non-tagged context probe, a GLMM testing whether group, stimulation and odor predicted percent time digging explained significantly more variance than the random effects ($\chi^2(7, N = 320) = 41.62$, $p < 0.0001$, $R^2 = 0.12$, nagelkerke-$R^2 = 0.38$; table S21). The EMMEANs (table S22 and fig. S3F) showed that, in the GFP group, contrasting wells, the mice dug significantly longer in the non-rewarded well during both stimulation OFF and ON sessions, as would be expected in the non-tagged context. Contrasting stimulation OFF and ON, there were no differences in percent time dug in either wells, showing that they dug equivalently in both sessions. In the ChR2 group, contrasting the wells, the mice dug significantly longer in the non-rewarded well during stimulation OFF, but reversed to digging longer in the rewarded well during stimulation ON. Contrasting stimulation ON and OFF, mice in the ChR2 group dug longer in the non-rewarded well during stimulation OFF, but reversed and dug longer in the rewarded well during stimulation ON. Collectively, these results provide evidence mice in the ChR2 group showed context-specific memory for the non-tagged context but shifted to a tagged-context-specific behavior during stimulation of the dentate gyrus context ensembles, suggesting that tagged context-encoding ensembles compose the ensembles encoding the context-odor pair associates, and their stimulation can drive (tagged) context-specific memory recall and guide behavior.

Table S21: GLMM evaluating the effect of group, stimulation and odor on percent time dug during non-tagged context probes

	time_dug				
Predictors	*Estimates*	*std. Error*	*CI*	*z value*	*p value*
(Intercept)	16.63 ***	2.60	11.52 – 21.75	6.40	**<0.001**
group [ChR2]	-7.39 *	3.68	-14.63 – -0.15	-2.01	**0.045**
stimulation [ON]	-3.24	2.06	-7.29 – 0.81	-1.57	0.117
odor [RE]	-7.62 ***	2.06	-11.67 – -3.56	-3.70	**<0.001**
group [ChR2] × stimulation [ON]	-1.33	2.91	-7.06 – 4.40	-0.46	0.648
group [ChR2] × odor [RE]	1.87	2.91	-3.86 – 7.60	0.64	0.521
stimulation [ON] × odor [RE]	1.42	2.91	-4.30 – 7.15	0.49	0.625
(group [ChR2] × stimulation [ON]) × odor [RE]	9.83 *	4.12	1.73 – 17.93	2.39	**0.018**
**Random Effects**					
σ^2	84.76				
τ_{00} mouse	37.13				
ICC	0.30				
N mouse	16				
Observations	320				
Marginal R^2 / Conditional R^2	0.119 / 0.387				
Deviance	2357.234				
AIC	2352.067				
log-Likelihood	-1166.034				

** p<0.05 ** p<0.01 *** p<0.001*

Table S22: EMMEANS of contrasts of interest in percent time dug during novel context probe

Group	*Odor*	*Stim*	*Contrast*	*Estimate*	*SE*	*df*	*lower.CI*	*upper.CI*	*t ratio*	*p value*
GFP	NON	.	OFF - ON	3.24	2.06	298	-2.71	9.18	1.57	0.1560
GFP	RE	.	OFF - ON	1.81	2.06	298	-4.13	7.76	0.88	0.4138
ChR2	NON	.	OFF - ON	4.57	2.06	298	-1.38	10.51	2.22	0.0467
ChR2	RE	.	OFF - ON	-6.69	2.06	298	-12.63	-0.74	-3.25	0.0078
GFP	.	OFF	NON - RE	7.62	2.06	298	1.67	13.56	3.70	0.0031
GFP	.	ON	NON - RE	6.19	2.06	298	0.25	12.14	3.01	0.0114
ChR2	.	OFF	NON - RE	5.74	2.06	298	-0.20	11.69	2.79	0.0168
ChR2	.	ON	NON - RE	-5.51	2.06	298	-11.46	0.43	-2.68	0.0188
.	NON	OFF	GFP - ChR2	7.39	3.68	24	-4.26	19.04	2.01	0.0839
.	NON	ON	GFP - ChR2	8.72	3.68	24	-2.93	20.37	2.37	0.0467
.	RE	OFF	GFP - ChR2	5.52	3.68	24	-6.13	17.17	1.50	0.1758

.	RE	ON	GFP - ChR2	-2.98	3.68	24	-14.63	8.67	-0.81	0.4255

The discrimination analysis of the non-tagged context probes further confirmed the percent time digging analysis. A GLMM testing the effect of group and stimulation on discrimination index was significant (χ^2(4, N = 160) = 41.86, $p < 0.0001$, $R^2 = 0.18$, nagelkerke-$R^2 = 0.26$) and the EMMEANS from the model showed that the GFP had a discrimination significantly below chance level (zero) during both stimulation conditions, whereas the ChR2 group had a discrimination significantly below chance during stimulation OFF that shifter to significantly above chance during stimulation ON (table S23). Contrasting stimulation conditions in the GFP group showed no difference in discrimination, whereas in the ChR2 group, there was a significantly higher discrimination during stimulation ON. Contrasting groups during stimulation OFF showed no discrimination difference, whereas during stimulation ON there was a higher discrimination in the ChR2 group (table S24 and fig. S3G). Collectively, these results provide further evidence that mice in the ChR2 group showed context-specific memory for the non-tagged context but shifted to a (tagged) context-specific behavior during stimulation of dentate gyrus context ensembles.

Table S23: EMMEANS comparing discrimination to chance (zero) across group and stimulation conditions during non-tagged context probes

stimulation	*group*	*emmean*	*SE*	*df*	*lower.CL*	*upper.CL*	*t.ratio*	*p value*
OFF	*GFP*	-0.32	0.11	30	-0.54	-0.10	-2.95	0.0082
ON	*GFP*	-0.38	0.11	30	-0.60	-0.16	-3.50	0.0030
OFF	*ChR2*	-0.46	0.11	30	-0.68	-0.23	-4.18	0.0009
ON	*ChR2*	0.24	0.11	30	0.02	0.47	2.23	0.0334

Table S24: EMMEANS of contrasts of interest in discrimination during non-tagged context probe

Group	*Stim*	*Contrast*	*Estimate*	*SE*	*df*	*lower.CI*	*upper.CI*	*t ratio*	*p value*
GFP	*.*	*OFF - ON*	0.06	0.13	137	-0.26	0.38	0.50	0.6206
ChR2	*.*	*OFF - ON*	-0.73	0.13	137	-1.05	-0.40	-5.69	<0.0001
.	*OFF*	*GFP - ChR2*	0.14	0.16	30	-0.28	0.56	0.86	0.5290
.	*ON*	*GFP - ChR2*	-0.65	0.16	30	-1.08	-0.23	-4.13	0.0005

Experiment 6: Stimulation of 'blocked context session' ensembles drives context-specific behavior in a novel context in the context-odor pair associate task

We tested whether photostimulation of neuronal ensembles tagged during a blocked context session, after the context-odor pair associate task training, could drive context-specific behavior in a novel context (fig. S4). Mice were microinjected with virus expressing RAM-ChR2 (*N* = 8) or RAM-GFP (*N* = 7) and trained in the context-odor pair associate task. After training, mice were removed from the DOX diet for 48 h and trained in either the black or white context (counterbalanced) using a blocked context session to tag the active ensembles of neurons. Mice were replaced on DOX diet following this training session. On the following three days, we performed a similar blocked context session, but in the non-tagged context to ensure equal context exposure, and two intermixed context sessions to ensure the behavior was not affected by the blocked context sessions. Mice were tested in two probe sessions in a novel context with photostimulation ON or OFF (fig. S4, A and B).

In the training data, the GLMM evaluating the effect of day, context and group on the likelihood of correct response was significant ($\chi 2(3, N = 2688) = 409.62$, $p < 0.0001$, $R2 = 0.33$, nagelkerke-$R2 = 0.34$; table S25). The likelihood of correct response was not affected by context, but significantly increased by a ratio of 1.39 per day, providing evidence for learning the context-odor pair associate task. The likelihood of correct response in the ChR2 group was lower at a ratio of 0.72 that of the GFP group, suggesting a slower learning in the ChR2 group. Nevertheless, both groups reached close to maximal performance in the context-odor pair associate task by day 10, which was maintained until day 15 (fig. S4C).

Table S25: GLMM testing context, day and group as predictors of (correct) response during training

	response				
Predictors	*Odds Ratios*	*std. Error*	*CI*	*z value*	*p value*
(Intercept)	1.30	0.18	0.99 – 1.71	1.88	0.060
group [ChR2]	0.72 *	0.10	0.55 – 0.94	-2.44	**0.015**
context [W]	0.83	0.09	0.66 – 1.03	-1.71	0.088
day	1.33 ***	0.02	1.29 – 1.38	16.78	**<0.001**
**Random Effects**					
σ^2	3.29				
$\tau_{00\ mouse}$	0.02				
ICC	0.01				
N_{mouse}	15				
Observations	2940				
Marginal R^2 / Conditional R^2	0.331 / 0.335				
Deviance	2083.515				
AIC	2093.515				
log-Likelihood	-1041.758				

* *p<0.05* ** *p<0.01* *** *p<0.001*

In the novel context probe, we tested whether photostimulation, odor and group predicted percent time digging. The GLMM yielded a significant model ($\chi 2(7, N = 300) = 45.38$, $p < 0.0001$,

R2 = 0.14, nagelkerke-R2 = 0.20; summarized in table S26). We tested the contrasts of interest using EMMEANS (table S27). Contrasting odor conditions, mice in the GFP group dug equivalently in the wells during both stimulation OFF and ON epochs, whereas mice the ChR2 group dug equivalently in the wells during stimulation OFF, but dug longer in the rewarded well during stimulation ON (fig. S4D). Contrasting stimulation conditions, the mice in the GFP group dug longer in both wells during stimulation ON than OFF, suggesting this group may have dug longer during the stimulation ON session overall. The mice in the ChR2 group did not dig differently in the non-rewarded well, but they dug longer in the rewarded well during stimulation ON. Contrasting the groups, the GFP group dug longer than the ChR2 group in the non-rewarded well during stimulation ON. No other differences were significant, although there was a generally higher percent time digging in the GFP group during stimulation ON. Taken together, these results suggest that photostimulation of dentate gyrus ensembles encoding a single-context-session can drive memory recall (and behavior) towards what would be expected in the context of the tagged session.

Table S26: GLMM evaluating the effect of group, stimulation and odor on percent time dug during novel context probes

	time_dug				
Predictors	*Estimates*	*std. Error*	*CI*	*z value*	*p value*
(Intercept)	3.67 *	1.66	0.40 – 6.94	2.21	**0.028**
group [ChR2]	-1.89	2.27	-6.37 – 2.58	-0.83	0.406
stimulation [ON]	7.42 ***	2.03	3.43 – 11.42	3.66	**<0.001**
odor [RE]	0.33	2.03	-3.67 – 4.32	0.16	0.872
group [ChR2] × stimulation [ON]	-6.58 *	2.78	-12.05 – -1.11	-2.37	**0.019**
group [ChR2] × odor [RE]	-0.93	2.78	-6.40 – 4.54	-0.34	0.737
stimulation [ON] × odor [RE]	-2.70	2.87	-8.35 – 2.95	-0.94	0.348
(group [ChR2] × stimulation [ON]) × odor [RE]	10.03 *	3.93	2.29 – 17.77	2.55	**0.011**
**Random Effects**					
σ^2	72.11				
$\tau_{00\ mouse}$	4.89				
ICC	0.06				
N_{mouse}	15				
Observations	300				
Marginal R^2 / Conditional R^2	0.143 / 0.198				
Deviance	2139.565				
AIC	2138.009				
log-Likelihood	-1059.004				

* p<0.05 ** p<0.01 *** p<0.001

Table S27: EMMEANS of contrasts of interest in percent time dug during novel context probe

Group	*Stim*	*Odor*	*Contrast*	*Estimate*	*SE*	*df*	*lower.CI*	*upper.CI*	*t ratio*	*p value*
GFP	*OFF*	*.*	*NON - RE*	-0.33	2.03	279	-6.19	5.54	-0.16	0.8715

GFP	*ON*	*.*	*NON - RE*	2.37	2.03	279	-3.50	8.23	1.17	0.4188
ChR2	*OFF*	*.*	*NON - RE*	0.60	1.90	279	-4.88	6.09	0.32	0.8567
ChR2	*ON*	*.*	*NON - RE*	-6.73	1.90	279	-12.21	-1.24	-3.54	0.0014
GFP	*.*	*NON*	*OFF - ON*	-7.42	2.03	279	-13.29	-1.56	-3.66	0.0014
GFP	*.*	*RE*	*OFF - ON*	-4.73	2.03	279	-10.59	1.14	-2.33	0.0495
ChR2	*.*	*NON*	*OFF - ON*	-0.84	1.90	279	-6.33	4.64	-0.44	0.8567
ChR2	*.*	*RE*	*OFF - ON*	-8.18	1.90	279	-13.66	-2.69	-4.31	0.0003
.	*OFF*	*NON*	*GFP - ChR2*	1.89	2.27	62	-4.87	8.66	0.83	0.6124
.	*ON*	*NON*	*GFP - ChR2*	8.47	2.27	62	1.71	15.24	3.73	0.0014
.	*OFF*	*RE*	*GFP - ChR2*	2.83	2.27	62	-3.94	9.59	1.24	0.4188
.	*ON*	*RE*	*GFP - ChR2*	-0.62	2.27	62	-7.39	6.14	-0.27	0.8567

The discrimination analysis of the novel context probes confirmed the percent time digging analysis. A GLMM testing the effect of group and stimulation on discrimination index was significant ($\chi2(3, N = 138) = 23.14$, $p < 0.0001$, $R2 = 0.15$, nagelkerke-$R2 = 0.16$). The EMMEANS showed that discrimination was at chance level (zero) in the GFP group during both stimulation conditions, whereas in the ChR2 group, discrimination was at chance level during stimulation OFF and increased significantly above chance during stimulation ON (table S28 and fig. S4E). Contrasting stimulation conditions in the ChR2 group, there was a significantly higher discrimination during stimulation ON, but no difference in the GFP group. Contrasting the groups, there was a higher discrimination in the ChR2 group during stimulation ON, but no difference during stimulation OFF (table S29 and fig. S4E). Taken together, these results provide evidence that photo-stimulation of (tagged) context-encoding dentate gyrus ensembles drove behavior from chance levels, expected in the novel context, to what would be expected in the tagged context, suggesting that dentate gyrus ensembles can drive context-specific memory recall (and behavior).

Table S28: EMMEANS comparing discrimination to chance (zero) across group and stimulation during novel context probes

stimulation	*group*	*emmean*	*SE*	*df*	*lower.CL*	*upper.CL*	*t.ratio*	*p value*
OFF	*GFP*	-0.01	0.12	46	-0.33	0.31	-0.11	0.9964
ON	*GFP*	0.00	0.13	43	-0.33	0.33	0.00	0.9964
OFF	*ChR2*	-0.19	0.11	39	-0.47	0.10	-1.72	0.1883
ON	*ChR2*	0.51	0.11	37	0.23	0.79	4.76	0.0001

Table S29: EMMEANS of contrasts of interest in discrimination during novel context probe

Group	*Stim*	*Contrast*	*Estimate*	*SE*	*df*	*lower.CI*	*upper.CI*	*t ratio*	*p value*
GFP	.	OFF - ON	-0.01	0.17	129	-0.45	0.42	-0.08	0.9395
ChR2	.	OFF - ON	-0.70	0.15	121	-1.07	-0.32	-4.73	<0.0001
.	OFF	GFP - ChR2	0.17	0.16	43	-0.26	0.60	1.05	0.3994
.	ON	GFP - ChR2	-0.51	0.17	40	-0.94	-0.08	-3.08	0.0074

Experiment 7: Calcium activity in CA1 ensembles during context-odor pair associate task training and context-only dentate gyrus ensemble chemogenetic interrogation

This experiment was a replication of Experiment 6 with the addition of a miniature microscope implanted over CA1 to record neuronal transient calcium activity (*N* = 6). Our goal was to replicate our findings in Experiment 4 and 6, and observe how neuronal activity in CA1 encoded the information about context and the context-odor pair associates, and how the manipulation of context-encoding dentate gyrus ensembles affect neuronal patterns during context-specific behavior (Fig. 3, A to D).

Table S30: Active fraction of cells over training and context

For each context in session over the final six training days, we quantified the number of cells detected on a given day divided by the total number of cells for that given mouse. We fit a repeated-measures ANOVA to detect if there were any differences in the number of active cells over training and between contexts. We did not fit a linear mixed effects model because we are not interested in getting a regression slope (we do not hypothesize this would increase with training). We found no significant main or interaction effect indicating that the number of active cells does not change across training days or context. Related to fig. S5B.

Predictors	F Value	Num DF	Den DF	p value
session	0.283	5	25	0.918
context	0.044	1	5	0.841
session x context	1.488	5	25	0.229

Table S31: Average transient rate over training and context

For each context in each session over the final six training days, we quantified the average transient rate for all active cells. We fit a repeated-measures ANOVA to detect if there were differences in the transient rate over training and between contexts. We found no significant

main or interaction effect indicating that the average transient rate does not change across training days or context. Related to fig. S5C.

Predictors	F Value	Num DF	Den DF	p value
session	0.876	5	25	0.511
context	0.066	1	5	0.807
session x context	0.739	5	25	0.601

Table S32: Active fraction of cells +/- C21 in novel context probe

To ask whether there is a significant difference in the number of detected cells in the novel context probe following C21 administration, we calculated the fraction of active cells for each mouse in the novel context probe in the VEH and C21 conditions and fit a linear mixed effects model on the data. We observed a non-significant negative slope, suggesting that there may be a small decrease in the number of cells detected with C21 administration in the novel context. Related to fig. S5B.

Predictors	*Coefficient*	*Std. Error*	*CI - 0.025*	*CI - 0.975*	*z value*	*p value*
Intercept	*0.897*	*0.325*	*0.261*	*1.534*	*2.762*	*0.006*
session	*-0.091*	*0.050*	*-0.188*	*0.006*	*-1.835*	*0.067*
Random Effects						
σ^2	*0.059*					
Group Var.	*0.007*					
ICC	*0.494*					
N mouse	*6*					
Observations	*12 mice-sessions*					
Marginal R^2 / Conditional R^2	*0.189 / 0.418*					
AIC	*-22.44*					
Log-likelihood	*15.22*					

Table S33: Active fraction of cells +/- C21 in non-tagged context probe

To ask whether there is a significant difference in the number of detected cells in the non-tagged context probe following C21 administration, we calculated the fraction of active cells for each mouse in the non-tagged context probe in the VEH and C21 conditions and fit a linear mixed effects model on the data. We observed a non-significant negative slope, suggesting that there may be a small decrease in the number of cells detected following C21 administration in the non-tagged context probe. Related to fig. S5B.

Predictors	Coefficient	Std. Error	CI - 0.025	CI - 0.975	z value	p value
Intercept	0.801	0.290	0.233	1.369	2.765	0.006
session	-0.064	0.034	-0.131	0.002	-1.891	0.059
Random Effects						
σ^2	0.032					
Group Var.	0.001					
ICC	0.282					
N mouse	6					
Observations	12 mice-sessions					
Marginal R^2 / Conditional R^2	0.189 / 0.418					
AIC	-88.597					
Log-likelihood	48.298					

Table S34: Average transient rate +/- C21 in novel context probe

To ask whether there is a significant difference in the average transient rate in the novel context probe depending on C21 administration, we calculated the fraction of active cells for each mouse in the novel context probe in the VEH and C21 conditions and fit a linear mixed effects model on the data. We observed a significant negative slope, suggesting that there is a significant decrease in the average transient rate with C21 administration in the novel context probe. Related to fig. S5C.

Predictors	*Coefficient*	*Std. Error*	*CI - 0.025*	*CI - 0.975*	*z value*	*p value*
Intercept	*0.076*	*0.010*	*0.055*	*0.097*	*7.248*	*0.000*

session	*-0.008*	*0.002*	*-0.011*	*-0.005*	*-4.898*	*0.000*
Random Effects						
σ^2	*5.26e-05*					
Group Var.	*0.000*					
ICC	*0.720*					
N mouse	*6*					
Observations	*12 mice-sessions*					
Marginal R^2 / Conditional R^2	*0.379 / 0.826*					
AIC	*-88.594*					
Log-likelihood	*48.297*					

Table S35: Average transient rate +/- C21 in non-tagged context probe

To ask whether there is a significant difference in the average transient rate in the non-tagged context probe depending on C21 administration, we calculated the fraction of active cells for each mouse in the non-tagged context probe in the VEH and C21 conditions and fit a linear mixed effects model on the data. We did not observe a significant difference in transient rate with C21 administration in the non-tagged context probe. Related to fig. S5C.

Predictors	Coefficient	Std. Error	CI - 0.025	CI - 0.975	z value	p value
Intercept	0.050	0.021	0.008	0.091	2.341	0.019
session	-0.003	0.002	-0.008	0.002	-1.245	0.213
Random Effects						
σ^2	2.24e-04					
Group Var.	0.000					
ICC	8.65e-09					
N mouse	6					
Observations	12 mice-sessions					
Marginal R^2 / Conditional R^2	0.123 / 0.123					

AIC	-88.597
Log-likelihood	48.298

A GLMM testing the effect of day and context in the likelihood of (correct) response was significant ($\chi 2(2, N = 1056) = 212.00$, $p < 0.0001$, $R2 = 0.35$, nagelkerke-$R2 = 0.39$; table S36). The likelihood of correct response was not significantly affected by context, but significantly increased by a ratio of 1.51 per day, showing evidence of learning context-odor pair associates across training days (Fig. 3E).

Table S36: GLMM evaluating the effect of context and day on (correct) response during context-odor pair associate training

	response				
Predictors	*Odds Ratios*	*std. Error*	*CI*	*z value*	*p value*
(Intercept)	0.52 *	0.14	0.30 – 0.89	-2.41	**0.016**
context [W]	0.93	0.16	0.67 – 1.30	-0.42	0.675
day	1.51 ***	0.05	1.41 – 1.62	12.10	**<0.001**
**Random Effects**					
σ^2	3.29				
$\tau_{00\ mouse}$	0.25				
ICC	0.07				
N_{mouse}	6				
Observations	1056				
Marginal R^2 / Conditional R^2	0.347 / 0.394				
Deviance	864.208				
AIC	872.208				
log-Likelihood	-432.104				

* *p<0.05* ** *p<0.01* *** *p<0.001*

In the novel context probe, a GLMM testing stimulation an odor conditions as predictors of percent time digging was significant ($\chi 2(3, N = 140) = 8.23$, $p = 0.0415$, $R2 = 0.05$, nagelkerke-$R2 = 0.10$; table S37). Testing the contrasts of interest via EMMEANS (table S38), we found that mice dug in the rewarded well for longer than the non-rewarded after C21 injection, but not after VEH. When comparing C21 to VEH injection directly, the mice did not dig differently in any well (Fig. 3F). These results replicate experiments biasing context-specific behavior using chemogenetic stimulation of dentate gyrus context-encoding ensembles.

Table S37: GLMM evaluating the effects of stimulation and odor on percent time digging during novel context probes

	time_dug				
Predictors	*Estimates*	*std. Error*	*CI*	*z value*	*p value*
(Intercept)	23.92 ***	4.90	14.23 – 33.61	4.88	**<0.001**
stimulation [C21]	-7.19	6.01	-19.08 – 4.69	-1.20	0.233
odor [RE]	0.38	6.09	-11.67 – 12.43	0.06	0.950
stimulation [C21] × odor [RE]	16.61	8.50	-0.20 – 33.41	1.95	0.053
**Random Effects**					
σ^2	631.29				
$\tau_{00\ mouse}$	32.55				
ICC	0.05				
N_{mouse}	6				
Observations	140				
Marginal R^2 / Conditional R^2	0.054 / 0.100				
Deviance	1300.669				
AIC	1293.014				
log-Likelihood	-640.507				

* *p<0.05* ** *p<0.01* *** *p<0.001*

Table S38: EMMEANS of contrasts of interest during the novel context probes

Odor	*Stim*	*Contrast*	*Estimate*	*SE*	*df*	*lower.CI*	*upper.CI*	*t ratio*	*p value*
RE	.	VEH - C21	7.19	6.01	131	-8.03	22.42	1.20	0.3114
NON	.	VEH - C21	-9.41	6.01	131	-24.64	5.81	-1.57	0.2395
.	VEH	NON - RE	-0.38	6.09	131	-15.82	15.05	-0.06	0.9501
.	C21	NON - RE	-16.99	5.92	131	-31.99	-1.99	-2.87	0.0192

The discrimination analysis of the novel context probes confirmed the percent time digging analysis. A GLMM testing the effect of stimulation (VEH or C21) on discrimination index was marginal (Χ2(2, N = 70) = 5.65, p = 0.0591, R2 = 0.05, nagelkerke-R2 = 0.09). The EMMEANS revealed a discrimination at chance level (zero) after VEH injection that shifted to significantly above chance level after C21 injections (table S39). When contrasting the conditions, there was a marginal significance for higher discrimination after C21 injection (emmean = 0.30, std. error = 0.16, df = 63, 95% CI = 0.01 - 0.61, t.ratio = 1.91, p = 0.0603). Together these results largely replicate our chemogenetic experiments and provide further evidence that stimulation of context encoding ensembles in the dentate gyrus can guide context-specific behavior in novel contexts.

Table S39: EMMEANS comparing discrimination to chance (zero) in the stimulation conditions during novel context probes

stimulation	*emmean*	*SE*	*df*	*lower.CL*	*upper.CL*	*t.ratio*	*p value*

VEH	-0.02	0.13	14	-0.29	0.26	-0.12	0.9046
C21	0.28	0.12	13	0.02	0.55	2.30	0.0396

In the non-tagged context probe, a GLMM testing the effect of stimulation and odor on percent time digging was significant ($\chi 2(3, N = 144) = 23.47$, $p < 0.0001$, $R2 = 0.12$, nagelkerke-$R2 = 0.29$; table S40). The EMMEANs showed a double dissociation in the odor and stimulation effects. Contrasting stimulation conditions, the mice dug significantly longer in the rewarded well after C21 injection than VEH. Following VEH injection, this pattern was reversed. Contrasting odors, the mice dug significantly longer in the non-rewarded well than in the rewarded one after VEH injection, but reversed that effect after C21 injection and dug longer in the rewarded well (table S41and Fig. 3G).

Table S40: GLMM evaluating the effects of stimulation and odor on percent time digging during novel context probes

	time_dug				
Predictors	*Estimates*	*std. Error*	*CI*	*z value*	*p value*
(Intercept)	39.83 ***	6.65	26.68 – 52.98	5.99	**<0.001**
stimulation [C21]	-27.39 ***	6.03	-39.32 – -15.46	-4.54	**<0.001**
odor [RE]	-19.67 **	6.03	-31.58 – -7.75	-3.26	**0.001**
stimulation [C21] × odor [RE]	40.00 ***	8.52	23.15 – 56.85	4.69	**<0.001**
**Random Effects**					
σ^2	653.64				
$\tau_{00\ mouse}$	156.27				
ICC	0.19				
N_{mouse}	6				
Observations	144				
Marginal R^2 / Conditional R^2	0.124 / 0.293				
Deviance	1349.522				
AIC	1340.719				
log-Likelihood	-664.359				

* *p<0.05* ** *p<0.01* *** *p<0.001*

Table S41: EMMEANS of contrasts of interest during the non-tagged context probes

Odor	*Stim*	*Contrast*	*Estimate*	*SE*	*df*	*lower.CI*	*upper.CI*	*t ratio*	*p value*
NON	.	VEH - C21	27.39	6.03	135	12.12	42.66	4.54	<0.0001
RE	.	VEH - C21	-12.61	6.03	135	-27.88	2.66	-2.09	0.0385
.	VEH	NON - RE	19.67	6.03	135	4.41	34.92	3.26	0.0019
.	C21	NON - RE	-20.33	6.03	135	-35.59	-5.08	-3.37	0.0019

The discrimination analysis of the non-tagged context probes confirmed the percent time digging analysis. A GLMM testing the effect of stimulation (VEH or C21) on discrimination index was significant ($R2 = 0.27$, $\chi 2(2, N = 67) = 21.96$, $p < 0.0001$, $R2 = 0.27$, nagelkerke-$R2 = 0.28$). The EMMEANS showed a discrimination significantly below chance level (zero) after VEH injection and above chance level after C21 injection (table S42). Additionally, there was a significantly higher discrimination after C21 injection than after VEH injection (emmean = 0.79, std. error = 0.16, df = 60, 95% CI = 0.48 - 1.11, t.ratio = 5.00, $p < 0.0001$). Taken together, these results provide further evidence that chemogenetic stimulation of tagged dentate gyrus ensembles encoding context can drive (tagged) context-specific behavior.

Table S42: EMMEANS comparing discrimination to chance (zero) in the stimulation conditions during non-tagged context probes

stimulation	*emmean*	*SE*	*df*	*lower.CL*	*upper.CL*	*t.ratio*	*p value*
VEH	-0.35	0.12	17	-0.63	-0.06	-2.94	0.0093
C21	0.45	0.12	15	0.16	0.74	3.85	0.0030

Table S43: Latency to first dig over training

We quantified the average time (in seconds) on each training day before mice first dug in a well (averaged over all trials within a training day). We fit a linear mixed effects model to the latency data over training (6 mice x 6 training days). We observed a significant negative slope, indicating that the latency to dig decreased as a function of training. Related to fig. S5E.

Predictors	Coefficient	Std. Error	CI - 0.025	CI - 0.975	z value	p value
Intercept	11.386	1.376	8.690	14.083	8.275	0.000
session	-0.878	0.397	-1.655	-0.101	-2.214	0.027
Random Effects						
σ^2	545.271					
Group Var.	2.710					
ICC	0.141					
N mouse	6					
Observations	36 mice-days					
Marginal R^2 / Conditional R^2	0.107 / 0.233					
AIC	215.222					

Log-likelihood	-103.6113

Table S44: Latency to first dig in novel context probe

We fit a linear mixed effects model to assess whether there was a significant difference in digging latency in the novel context in the VEH or C21 condition. The VEH condition is coded as 0 and C21 is coded as 1. We observed a significant negative slope indicating that we observed a significant decrease in digging latency with C21 administration. Related to fig. S5E.

Predictors	Coefficient	Std. Error	CI - 0.025	CI - 0.975	z value	p value
Intercept	20.669	2.722	15.33	26.004	7.593	0.000
session	-7.628	3.245	-13.989	-1.267	-2.350	0.019
Random Effects						
σ^2	294.0699					
Group Var.	12.866					
ICC	0.289					
N mouse	6					
Observations	36 mice-days					
Marginal R^2 / Conditional R^2	0.263 / 0.476					
AIC	87.065					
Log-likelihood	-39.532					

Table S45: Latency to first dig in non-tagged context probe

We fit a linear mixed effects model to assess whether there was a significant difference in digging latency in the non-tagged context probe in the VEH or C21 condition. The VEH condition is coded as 0 and C21 is coded as 1. We observed a non-significant positive slope, suggesting that there may be a small increase in digging latency with C21 administration in the non-tagged probe. Related to fig. S5E.

Predictors	Coefficient	Std. Error	CI - 0.025	CI - 0.975	z value	p value
Intercept	8.091	4.348	-0.431	16.613	1861	0.063

session	10.106	5.525	-0.723	20.934	1.829	0.067
Random Effects						
σ^2	921.282					
Group Var.	21.864					
ICC	0.192					
N mouse	6					
Observations	36 mice-days					
Marginal R^2 / Conditional R^2	0.197 / 0.351					
AIC	98.602					
Log-likelihood	-45.301					

Table S46: Between-context PV distance over training

To quantify how the contextual representation changes with experience, we calculated the average pairwise cosine distance between the entire trial (excluding time in reward zones) population vectors (PVs) of average fluorescence observed in different contexts and divided that by the average pairwise cosine distance between all PVs within the same context. We calculated the chance level by randomizing the context identity of each population vector. We then fit a linear mixed effects model on the data and observed a significant interaction between shuffle and session indicating an increasing difference between the actual PV distance vs. chance levels. Posthoc tests consisted of pairwise mixed effects models between shuffle and actual on each day. The p-values were corrected using a Holm-Sidak stepdown procedure. Related to Fig. 3I.

Predictors	Coefficient	Std. Error	CI - 0.025	CI - 0.975	z value	p value
Intercept	1.127	0.025	1.078	1.176	45.076	0.000
shuffle	-0.123	0.035	-0.191	-0.056	-3.564	0.000
session	0.030	0.008	0.014	0.046	3.718	0.000
shuffle x session	-0.032	0.011	-0.054	-0.009	-2.779	0.005
Random Effects						
σ^2	0.485					
Group Var.	0.000					

ICC	0.022
N mouse	6
Observations	72 mice - shuffles
Marginal R^2 / Conditional R^2	0.626/0.634
AIC	-140.926
Log-likelihood	76.463

Table S47: Between-context PV distance over training posthoc comparisons

Posthoc comparisons for table S46. Posthoc tests consisted of pairwise mixed effects models between shuffle and actual on each day. The p-values were corrected using a Holm-Sidak stepdown procedure. Related to Fig. 3I.

Group	Estimate	Std. Err.	lowerCI	upperCI	z	p	p-adjusted	reject null
Day 5, shuffle - no shuffle	-0.087	0.049	-0.183	0.008	-1.785	0.074	0.074	False
Day 6, shuffle - no shuffle	-0.192	0.050	-0.290	-0.094	-3.853	0.0001	0.0003	True
Day 7, shuffle - no shuffle	-0.262	0.047	-0.353	-0.0813	-5.602	0.000	0.000	True
Day 8, shuffle - no shuffle	-0.170	0.062	-0.292	-0.048	-2.736	0.006	0.012	True

Day 9, shuffle - no shuffle	-0.224	0.031	-0.286	-0.163	-7.176	0.000	0.000	True
Day 10, shuffle - no shuffle	-0.300	0.044	-0.387	-0.213	-6.768	0.000	0.000	True

Table S48: Latent-space conditional probability distribution distance over training (5D-PCA)

Using a different method, we sought to quantify the experience-dependent separation of PVs by context. For details about the procedure, see Methods. Briefly, for each day during training, we reduced the dimensionality of the population activity over an entire day using PCA to three dimensions. For each context, we estimated a probability distribution over the 5d latent space using kernel density estimation, and calculated a distribution distance. We repeated this procedure while randomizing the context-identity of each PV to calculate chance levels of distribution distance. We fit a linear mixed effects model on the data and observed a significant interaction effect, suggesting that relative to chance levels (shuffle) the distribution distance increases with experience. Related to Fig. 3J.

Predictors	Coefficient	Std. Error	CI - 0.025	CI - 0.975	z value	p value
Intercept	0.475	0.053	0.372	0.578	9.047	0.000
shuffle	-0.267	0.062	-0.389	-0.145	-4.294	0.000
session	0.044	0.015	0.015	0.072	3.022	0.000
shuffle x session	-0.042	0.021	-0.083	-0.002	-2.062	0.039
Random Effects						
σ^2	1.495					
Group Var.	0.005					
ICC	0.183					
N mouse	6					
Observations	72 mice - shuffles					
Marginal R^2 /	0.584/0.660					

Conditional R^2	
AIC	-50.25
Log-likelihood	31.126

Table S49: Latent-space conditional probability distribution distance over training (3D-PCA) posthoc comparisons

Posthoc comparisons for table S48. Posthoc tests consisted of pairwise mixed effects models between shuffle and actual on each day, p-values were corrected using a Holm-Sidak stepdown procedure. Related to Fig. 3J.

Group	Estimate	Std. Err.	lowerCI	upperCI	z	p	p-adjusted	reject null
Day 5, shuffle - no shuffle	-0.220	0.049	-0.315	-0.124	-4.512	0.000	0.000	True
Day 6, shuffle - no shuffle	-0.360	0.116	-0.587	-0.133	-3.111	0.001	0.001	True
Day 7, shuffle - no shuffle	-0.421	0.117	-0.651	-0.192	-3.607	0.000	0.000	True
Day 8, shuffle - no shuffle	-0.322	0.089	-0.497	-0.147	-3.614	0.000	0.000	True
Day 9, shuffle - no shuffle	-0.395	0.071	-0.534	-0.256	-5.573	0.000	0.000	True

Day 10, shuffle - no shuffle	-0.515	0.104	-0.719	-0.311	-4.955	0.000	0.000	True

Table S50: Within-context PV distance correlation to total distance traveled

We subsequently asked whether the within-context PV distance effect could be dependent on how the mice engage with the task. We calculated the total distance covered during each trial as a measure of mouse engagement level with a larger distance traveled indicating that mice spent more time engaging in non-task behaviors. We fit a linear mixed effects model with distance traveled as an independent variable to see if it correlated with the measured PV distance. We found no significant effect, suggesting that mouse behavior is not trivially affecting this measurement.

Predictors	Coefficient	Std. Error	CI - 0.025	CI - 0.975	z value	p value
Intercept	1.213	0.093	1.030	1.396	13.016	0.000
distance	-0.000	0.001	-0.002	0.002	-0.168	0.866
Random Effects						
σ^2	0.582					
Group Var.	0.000					
ICC	0.011					
N mouse	6					
Observations	36 mice-days					
Marginal R^2 / Conditional R^2	0.00082/0.011					
AIC	-37.569					
Log-likelihood	22.784					

Table S51: Latent-space conditional probability distribution distance correlation with total distance traveled

Similar to table S50 but with latent-space distribution distance as the dependent variable.

Predictors	Coefficient	Std. Error	CI - 0.025	CI - 0.975	z value	p value
Intercept	0.548	0.158	0.238	0.858	3.461	0.001
distance	0.000	0.002	-0.003	0.004	0.259	0.796
Random Effects						
σ^2	1.254					
Group Var.	0.016					
ICC	0.290					
N mouse	6					
Observations	36 mice-days					
Marginal R^2 / Conditional R^2	0.0016/0.291					
AIC	1.297					
Log-likelihood	3.351					

Table S52: Overlap in cells identified over training with training day 10

Since we base our subsequent analyses on comparison of different sessions to the final training day (day 10), we first quantified whether the degree of neural overlap (number of cells detected in session X and day 10 divided by number of cells detected in session X) between different sessions in training change across training days and context. We fit a repeated-measures ANOVA to detect differences in the degree of neural overlap. We did not fit a linear mixed effects model because we are not interested in getting a regression slope (we do not hypothesize this would increase with training). We found no significant main or interaction effect indicating that the degree of neural overlap does not change across training days or context. Related to fig. S6A.

Predictors	F Value	Num DF	Den DF	p value
session	0.5677	4	20	0.689
context	1.9718	1	5	0.219

session x context	-0.992	4	20	1.000

Table S53: Overlap in cells identified in novel context probe with training day 10

To ask whether there is a significant difference in the degree of neural overlap with training day 10 in the novel context probe following C21 administration, we calculated the overlap in detected cells with day 10 for each mouse in the novel context probe in the VEH and C21 conditions and fit a linear mixed effects model on the data. We did not observe a significant effect indicating that C21 administration does not affect the degree of overlap in the detected cells with day 10 in the novel context probe. Related to fig. S6A.

Predictors	Coefficient	Std. Error	CI - 0.025	CI - 0.975	z value	p value
Intercept	0.379	0.123	0.139	0.620	3.091	0.002
session	0.007	0.016	-0025	0.039	0.444	0.657
Random Effects						
σ^2	0.0049					
Group Var.	0.022					
ICC	0.963					
N mouse	6					
Observations	12 mice-sessions					
Marginal R^2 / Conditional R^2	0.00064 / 0.964					
AIC	-19.41					
Log-likelihood	13.7					

Table S54: Overlap in cells identified in non-tagged context probe with training day 10

To ask whether there is a significant difference in the degree of neural overlap with training day 10 in the non-tagged context probe depending on C21 administration, we calculated the overlap in detected cells with day 10 for each mouse in the non-tagged context probe in the VEH and C21 conditions and fit a linear mixed effects model on the data. We did not observe a significant effect indicating that C21 administration does not affect the degree of overlap in the detected cells with day 10 in the non-tagged context probe. Related to fig. S6A.

Predictors	Coefficient	Std. Error	CI - 0.025	CI - 0.975	z value	p value
Intercept	0.669	0.297	0.086	1.251	2.249	0.025
session	-0.025	0.034	-0.092	0.042	-0.744	0.457
Random Effects						
σ^2	0.022					
Group Var.	0.023					
ICC	0.866					
N mouse	6					
Observations	12 mice-sessions					
Marginal R^2 / Conditional R^2	0.0066 / 0.867					
AIC	-9.962					
Log-likelihood	8.981					

Table S55: PV similarity in training

This analysis calculates PVs of average fluorescence for each context on each training day, and for each context and day we calculate the PV similarity to PVs from each context on day 10 of training. There is a significant interaction of context type (corr_type) (same vs. different context) across training, indicating that over training, the representation of context by average activity becomes more distinct from the other context. For this model we excluded the reference day 10 since the same context category is a similarity calculation to itself. Posthoc tests consisted of pairwise mixed effects models between the two contexts on each day, p-values were corrected using a Holm-Sidak stepdown procedure. Related to Fig. 4B.

Predictors	Coefficient	Std. Error	CI - 0.025	CI - 0.975	z value	p value
Intercept	0.069	0.065	-0.058	0.195	1.067	0.286
ctx_similarity	0.118	0.076	-0.031	0.268	1.557	0.120
session	-0.001	0.022	-0.044	0.042	-0.054	0.957
ctx_similarity x session	0.061	0.031	0.001	0.122	1.981	0.048
Random Effects						

σ^2	3206.059
Group Var.	0.005
ICC	0.004
N mouse	6
Observations	2556 cells - contexts
Marginal R^2 / Conditional R^2	0.0142/0.018
AIC	7853.807
Log-likelihood	-3920.903

Table S56: PV similarity in training posthoc comparisons

Posthoc comparisons for table S55. Posthoc tests consisted of pairwise mixed effects models between the two contexts on each day, p-values were corrected using a Holm-Sidak stepdown procedure. Related to Fig. 4B.

Group	Estimate	Std. Err.	lowerCI	upperCI	z	p	p-adjusted	reject null
Day 5, Same ctx - diff ctx	0.165	0.118	-0.065	0.397	1.402	0.160	0.295	False
Day 6, Same ctx - diff ctx	0.095	0.090	-0.080	0.271	1.065	0.287	0.295	False
Day 7, Same ctx - diff ctx	0.289	0.083	0.127	0.451	3.507	0.00045	0.0022	True
Day 8,	0.261	0.084	0.097	0.426	3.118	0.0018	0.0054	True

Same ctx - diff ctx								
Day 9, Same ctx - diff ctx	0.383	0.110	0.168	0.598	3.499	0.00046	0.0022	True

Table S57: PV similarity in novel context probe

For each session (VEH, C21) in the novel context probe, we tested differences between correlation to the tagged or non-tagged context using pairwise mixed effects models (*N* = 6 mice, *n* = 960 cells), p-values were corrected using a Holm-Sidak stepdown procedure. Related to Fig. 4C.

Group	Estimate	Std. Err.	lowerCI	upperCI	z	p	p-adjusted	reject null
-C21, Tagged – Non-tagged context	0.107	0.093	-0.0751	0.290	1.154	0.248	0.248	False
+C21, Tagged – Non-tagged context	0.195	0.115	-0.0401	0.411	1.612	0.107	0.107	False

Table S58: PV similarity in non-tagged context probe

For each session (VEH, C21) in the non-tagged context probe, we tested differences between correlation to the tagged or non-tagged context using pairwise mixed effects models (*N* = 6 mice, *n* = 826 cells), p-values were corrected using a Holm-Sidak stepdown procedure. Related to Fig. 4D.

Group	Estimate	Std. Err.	lowerCI	upperCI	z	p	p-adjusted	reject null
-C21, Tagged – Non-tagged context	-0.214	0.105	-0.419	-0.008	-2.042	0.0411	0.0551	True
+C21, Tagged – Non-tagged context	-0.259	0.118	-0.490	-0.028	-2.197	0.0279	0.0551	True

Table S59: NCM similarity in training

This analysis calculates normalized correlation matrices over an entire trial for each context on each training day. For each cell, we calculated its normalized correlation to every other cell on each context and day, and calculated the similarity to the NCM row corresponding to that cell from each context on day 10 of training. There is a significant interaction of context (same or different context) and session (training day) across training, indicating that over training, the representation of context by average activity becomes more distinct. For this model we excluded the reference day 10 since the same context category is a similarity calculation to itself. Note, in this analysis, "same context" is coded numerically as 0, therefore a negative coefficient for the ctx_similarity x session interaction indicates a greater similarity of pairwise correlations between the same contexts versus different contexts. Related to Fig. 4F.

Predictors	Coefficient	Std. Error	CI - 0.025	CI - 0.975	z value	p value
Intercept	0.083	0.037	0.010	0.155	2.239	0.025
ctx_similarity	-0.014	0.019	-0.050	0.023	-0.720	0.427
session	0.022	0.006	0.011	0.033	3.771	0.000
ctx_similarity x session	-0.016	0.008	-0.032	-0.000	-1.991	0.046
Random Effects						
σ^2	27.56					
Group Var.	0.006					
ICC	0.162					

N mouse	6
Observations	886 cells - sessions
Marginal R^2 / Conditional R^2	0.025/0.184
AIC	-525.775
Log-likelihood	268.88

Table S60: NCM similarity in training posthoc comparisons

Posthoc comparisons for table S59. Posthoc tests consisted of pairwise mixed effects models between the two contexts on each day, p-values were corrected using a Holm-Sidak stepdown procedure. Related to Fig. 4F.

Group	Estimate	Std. Err.	lowerCI	upperCI	z	p	p-adjusted	reject null
Day 5, Same ctx - diff ctx	-0.015	0.021	-0.056	0.024	-0.760	0.447	0.447	False
Day 6, Same ctx - diff ctx	-0.031	0.025	-0.079	0.017	-1.268	0.204	0.367	False
Day 7, Same ctx - diff ctx	-0.037	0.025	-0.086	0.011	-1.483	0.138	0.359	False
Day 8, Same ctx - diff ctx	-0.067	0.032	-0.130	-0.005	-2.121	0.033	0.128	False

Day 9, Same ctx - diff ctx	-0.080	0.028	-0.136	-0.0248	-2.834	0.004	0.022	True

Table S61: NCM similarity in novel context probe

For each session (VEH, C21) in the novel context probe, we tested differences between NCM correlation to the tagged or non-tagged context using pairwise mixed effects models (N = 6 mice, n = 960 cells), p-values were corrected using a Holm-Sidak stepdown procedure. Related to Fig. 4G.

Group	Estimate	Std. Err.	lowerCI	upperCI	z	p	p-adjusted	reject null
-C21, Tagged – Non-tagged context	0.0042	0.018	-0.031	0.040	0.235	0.814	0.922	False
+C21, Tagged – Non-tagged context	-0.0081	0.023	-0.053	0.0367	-0.357	0.720	0.922	False

Table S62: NCM similarity in non-tagged context probe

For each session (VEH, C21) in the non-tagged context probe, we tested differences between NCM correlation to the tagged or non-tagged context using pairwise mixed effects models (N = 6 mice, n = 824 cells), p-values were corrected using a Holm-Sidak stepdown procedure. Related to Fig. 4H.

Group	Estimate	Std. Err.	lowerCI	upperCI	z	p	p-adjusted	reject null
-C21, Tagged – Non-tagged	-0.075	0.020	-0.113	-0.036	-3.817	0.0001	0.0002	True

context								
+C21, Tagged – Non-tagged context	-0.093	0.029	-0.150	-0.036	-3.218	0.001	0.001	True

Table S63: Ensembles in training

For each context on training day 10, we extracted significant ensembles from population activity (see methods for details). We then calculated the ensemble activation rate for ensembles extracted from either the same or the opposite context for each context on previous training days. We found a significant effect of context similarity (same or different context) and session (training day) indicating that the ensemble structure between contexts becomes more distinct with experience. For this model we excluded the reference day 10. Related to Fig. 5C.

Predictors	Coefficient	Std. Error	CI - 0.025	CI - 0.975	z value	p value
Intercept	0.018	0.004	0.010	0.026	4.514	0.000
context	0.004	0.004	-0.003	0.012	1.162	0.245
session	0.001	0.001	-0.001	0.003	1.214	0.225
context x session	0.003	0.001	0.001	0.006	2.392	0.017
Random Effects						
σ^2	1.542					
Group Var.	0.000					
ICC	0.036					
N mouse	6					
Observations	1152 trials – contexts					
Marginal R^2 / Conditional R^2	0.042/0.077					
AIC	-4322.14					
Log-likelihood	2167.07					

Table S64: Ensembles in training posthoc comparisons

Posthoc comparisons for table S63. Posthoc tests consisted of pairwise mixed effects models between the two contexts on each day, p-values were corrected using a Holm-Sidak stepdown procedure. Related to Fig. 5C.

Group	Estimate	Std. Err.	lowerCI	upperCI	z	p	p-adjusted	reject null
Day 5, Same ctx - diff ctx	0.0045	0.003	-0.0014	0.010	1.489	0.136	0.136	False
Day 6, Same ctx - diff ctx	0.007	0.004	0.000	0.0156	1.980	0.047	0.105	False
Day 7, Same ctx - diff ctx	0.007	0.004	0.000	0.014	2.252	0.024	0.093	False
Day 8, Same ctx - diff ctx	0.013	0.007	0.000	0.026	2.091	0.036	0.105	False
Day 9, Same ctx - diff ctx	0.022	0.008	0.007	0.037	2.986	0.003	0.018	True
Day 10, Same ctx - diff ctx	0.015	0.003	0.009	0.022	4.738	0.000	0.000	True

Table S65: Ensemble activation in novel context probe

For each context on training day 10, we extracted significant ensembles (see methods for details). For each trial in either session (VEH or C21), we calculated the average ensemble activation rate for ensembles extracted from each context. We then normalized the context-specific ensemble activation rate to the total ensemble activation rate (ensemble activation ratio) to control for differing levels of neuronal overlap and total activity. We excluded any trials where the total ensemble activation rate was zero. These occurred primarily in one mouse with relatively few neurons detected, so this mouse had no trials in the novel context with significant ensemble activations from either context. We observed a significant effect of C21 on ensemble activation ratio, indicating that dentate gyrus context ensemble activation increased the relative frequency of tagged-context ensemble activation during the novel context probe. Related to Fig. 5D.

Predictors	Coefficient	Std. Error	CI - 0.025	CI - 0.975	z value	p value
Intercept	0.522	0.043	0.437	0.607	12.02	0.000
context	-0.043	0.061	-0.164	0.077	-0.707	0.480
C21	-0.164	0.061	-0.283	-0.045	-2.965	0.007
context x C21	0.328	0.086	0.159	0.497	3.811	0.000
Random Effects						
σ^2	6.011					
Group Var.	0.000					
ICC	0.000					
N mouse	5					
Observations	114 trials - contexts					
Marginal R^2 / Conditional R^2	0.167/0.167					
AIC	0.073					
Log-likelihood	5.963					

Table S66: Ensemble activation in novel context probe posthoc comparisons

Posthoc comparisons for table S65. Posthoc tests consisted of pairwise mixed effects models between the two contexts on each day, p-values were corrected using a Holm-Sidak stepdown procedure. Related to Fig. 5D.

Group	Estimate	Std. Err.	lowerCI	upperCI	z	p	p-adjusted	reject null
-C21, Tagged – Non-tagged context	-0.043	0.051	-0.144	0.057	-0.845	0.398	0.398	False
+C21, Tagged – Non-tagged context	0.284	0.070	0.146	0.422	4.045	0.000	0.000	True

Table S67: Ensemble activation in non-tagged context probe

For each context on training day 10, we extracted significant ensembles (see methods for details). For each trial in either session (VEH or C21), we calculated the average ensemble activation rate for ensembles extracted from each context. We then normalized the context-specific ensemble activation rate to the total ensemble activation rate (ensemble activation ratio) to control for differing levels of neuronal overlap and total activity. We excluded any trials where the total ensemble activation rate was zero. We observed a significant effect of C21 on ensemble activation ratio, indicating that dentate gyrus context ensemble activation increased the relative frequency of tagged-context ensemble activation during the non-tagged context probe. Related to Fig. 5E.

Predictors	Coefficient	Std. Error	CI - 0.025	CI - 0.975	z value	p value
Intercept	1.029	0.176	0.684	1.374	5.850	0.000
context	-1.058	0.249	-1.545	-0.570	-4.252	0.000
C21	-0.203	0.068	-0.337	-0.069	-2.963	0.003
context x C21	0.405	0.097	0.216	0.595	4.190	0.000
Random Effects						
σ^2	11.12					
Group Var.	0.000					
ICC	0.000					

N mouse	6
Observations	138 trials - contexts
Marginal R^2 / Conditional R^2	0.116/0.116
AIC	56.08
Log-likelihood	-22.042

Table S68: Ensemble activation in non-tagged context probe posthoc comparisons

Posthoc comparisons for table S67. Posthoc tests consisted of pairwise mixed effects models between the two contexts on each day, p-values were corrected using a Holm-Sidak stepdown procedure. Related to Fig. 5E.

Group	Estimate	Std. Err.	lowerCI	upperCI	z	p	p-adjusted	reject null
-C21, Tagged – Non-tagged context	-0.246	0.064	-0.372	-0.120	-3.842	0.000	0.000	True
+C21, Tagged – Non-tagged context	0.158	0.073	0.014	0.302	2.164	0.030	0.030	True

Table S69: Number of ensembles identified over training by context

Using the ensemble detection procedure described in the Methods, we extracted ensembles from each context on each training day and calculated the number of ensembles detected relative to the number of detected cells. We then asked whether there were any differences with context or experience. We fit a linear mixed effects model and observed no significant main effect of context or interaction effect, suggesting that context does not affect the number of ensembles detected. We did observe a significant positive slope for session, suggesting that the number of ensembles detected increases with experience, although the magnitude of this effect is small. Related to fig. S6B.

Predictors	Coefficient	Std. Error	CI - 0.025	CI - 0.975	z value	p value
Intercept	0.190	0.013	0.165	0.215	14.857	0.000
context	0.02	0.011	-0.002	0.041	1.799	0.072
session	0.004	0.003	-0.001	0.009	1.647	0.100
context x session	-0.006	0.004	-0.013	0.001	-1.596	0.111
Random Effects						
σ^2	0.046					
Group Var.	0.001					
ICC	0.471					
N mouse	6					
Observations	72 mice – contexts					
Marginal R^2 / Conditional R^2	0.027/0.485					
AIC	-292.79					
Log-likelihood	152.39					

Table S70: Ensemble activations coincide with synchronous calcium events

For each context and training day, we calculated time courses of significant ensemble activations and significant synchronous calcium events (or SCEs). We calculated the correlation between the vector indicating time bins with significant ensemble activation and the vector indicating time bins with significant SCE activity. We conducted 100 circular shuffles to get chance level correlations. We then averaged the correlation within mice, training day, and context. Shuffled data is coded as 1, so a negative slope for the shuffle indicates that correlation values are higher for actual data. Related to Fig. 5G. .

Predictors	Coefficient	Std. Error	CI - 0.025	CI - 0.975	z value	p value
Intercept	0.133	0.006	0.122	0.144	23.032	0.000
shuffle	-0.134	0.007	-0.147	-0.121	-19.688	0.000

Random Effects	
σ^2	0.235
Group Var.	0.000
ICC	0.035
N mouse	6
Observations	144 context-training days
Marginal R^2 / Conditional R^2	0.723/0.733
AIC	-500.825
Log-likelihood	254.412

Table S71: Probability of observing significant ensemble activation event during an SCE is greater than chance level

For each context and training day, we calculated time courses of significant ensemble activations and significant synchronous calcium events (or SCEs). We calculated the probability that each timebin shows a significant SCE given it is shows significant ensemble activation. We conducted 100 circular shuffles to get chance level co-occurrence probabilities. We then averaged the correlation within mice, training day, and context. Shuffled data is coded as 1, so a negative slope for the shuffle indicates that correlation values are higher for actual data. Related to Fig. S6D.

Predictors	Coefficient	Std. Error	CI - 0.025	CI - 0.975	z value	p value
Intercept	0.120	0.015	0.091	0.149	8.240	0.000
shuffle	-0.068	0.008	-0.084	-0.052	-8.459	0.000
Random Effects						
σ^2	0.325					
Group Var.	0.001					
ICC	0.314					
N mouse	6					
Observations	144 context-training days					

Marginal R^2 / Conditional R^2	0.255/0.489
AIC	-440.182
Log-likelihood	224.09

Table S72: Quantification of peri-SCE speed timecourse

Relative speed was averaged within three time bins (-2 to -1s: Pre, -0.5 to 0.5s: During, 1 to 2s: Post), and then averaged across SCEs within a single training day. Linear mixed-effects model was fit to the groups indicated below. Related to fig. S6E.

Group	Estimate	Std. Err.	lowerCI	upperCI	z	p
Pre-During	0.035	0.039	-0.042	0.112	0.893	0.372
During-Post	0.101	0.040	0.022	0.179	2.522	0.012

Table S73: Quantification of peri-SCE sniffing timecourse

Relative sniffing probability was averaged within three time bins (-2 to -1s: Pre, -0.5 to 0.5s: During, 1 to 2s: Post), and then averaged across SCEs within a single training day. Linear mixed-effects model was fit to the groups indicated below. Related to fig. S6F.

Group	Estimate	Std. Err.	lowerCI	upperCI	z	p
Pre-During	-0.196	0.050	-0.294	-0.098	-3.927	0.000
During-Post	-0.184	0.054	-0.290	-0.077	-3.386	0.001

Table S74: Experimental design for context-odor paired associate experiments

*****tagging session

Exp #	virus	days -2 - 0	days 1-4 training	days 5-10 training	days 11-12	day 13	days 14-15	days 16-17
2	-	hab & shaping	phase 1	phase 2	probe	-	-	-
3	hM4D	hab* & shaping	phase 1	phase 2	tagged probes	-	-	-
4	hM3D	hab* & shaping	phase 1	phase 2	novel probes	interleaved session	non-tagged probes	-
5	ChR2	hab* & shaping	phase 1	phase 2	novel probes	interleaved session	non-tagged probes	-
6	ChR2	hab & shaping	phase 1	phase 2	blocked session*	blocked session	interleaved sessions	novel probes
7	hM3D	hab* & shaping	phase 1	phase 2	novel probes	interleaved session	non-tagged probes	-

SUPPLEMENTARY FIGURES

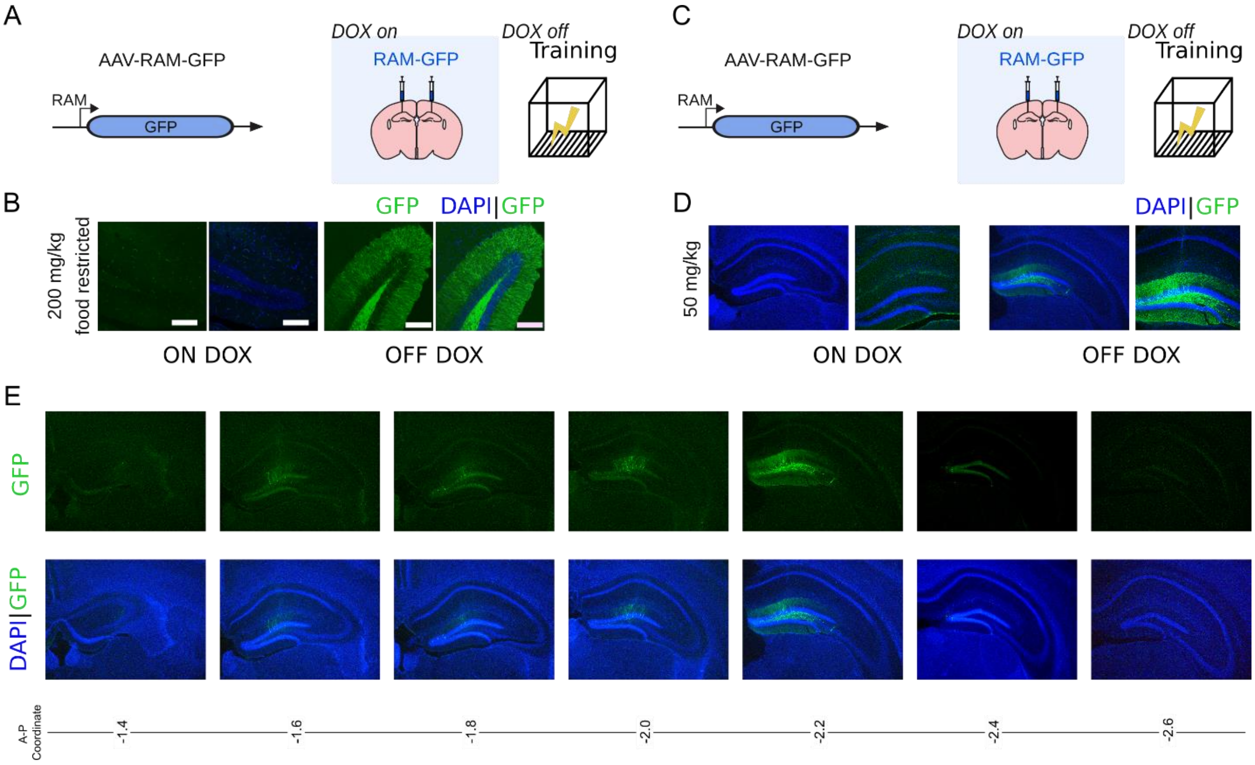

Figure S1. Determination of DOX concentration for task-specific neuronal tagging using RAM vector under food restriction. (**A**) AAV-RAM-GFP virus construct was microinjected in the dentate gyrus. Removal of DOX (200 mg/kg) from diet in food-restricted mice permitted tagging of dentate gyrus ensembles active during contextual fear conditioning. (**B**) GFP expression in dentate gyrus 24 h after contextual fear conditioning, either “ON” or “OFF” DOX diet (left and right). DOX suppressed GFP expression under food restriction in DOX ON condition but allowed strong GFP expression 48 h after DOX removal from diet (DOX OFF). GFP = green, DAPI = blue, scale bars = 100 µm. (**C**) AAV-RAM-GFP virus construct was microinjected in the dentate gyrus. Removal of DOX (50 mg/kg) from diet permitted tagging of dentate gyrus ensembles active during contextual fear conditioning in non-food restricted mice. (**D**) (*top*) Low and high magnification examples of robust activity-induced GFP expression restricted to dentate gyrus. (*bottom*) Low and high magnification examples from mice which were subjected to fear conditioning while being maintained on a DOX diet. No GFP expression was seen, indicating that the RAM tagging system is not leaky. (**E**) Activity-dependent labeling of dentate gyrus ensembles across the anterior-posterior axis. GFP expression via the RAM tagging system was typically spanned 1 mm of the anterior-posterior axis. Within sections showing maximal viral expression, approximately 3% of DG neurons expressed GFP (3.5% +/- 0.39, *N* = 3), with no expression observed in CA3 or CA1 regions of the hippocampus.

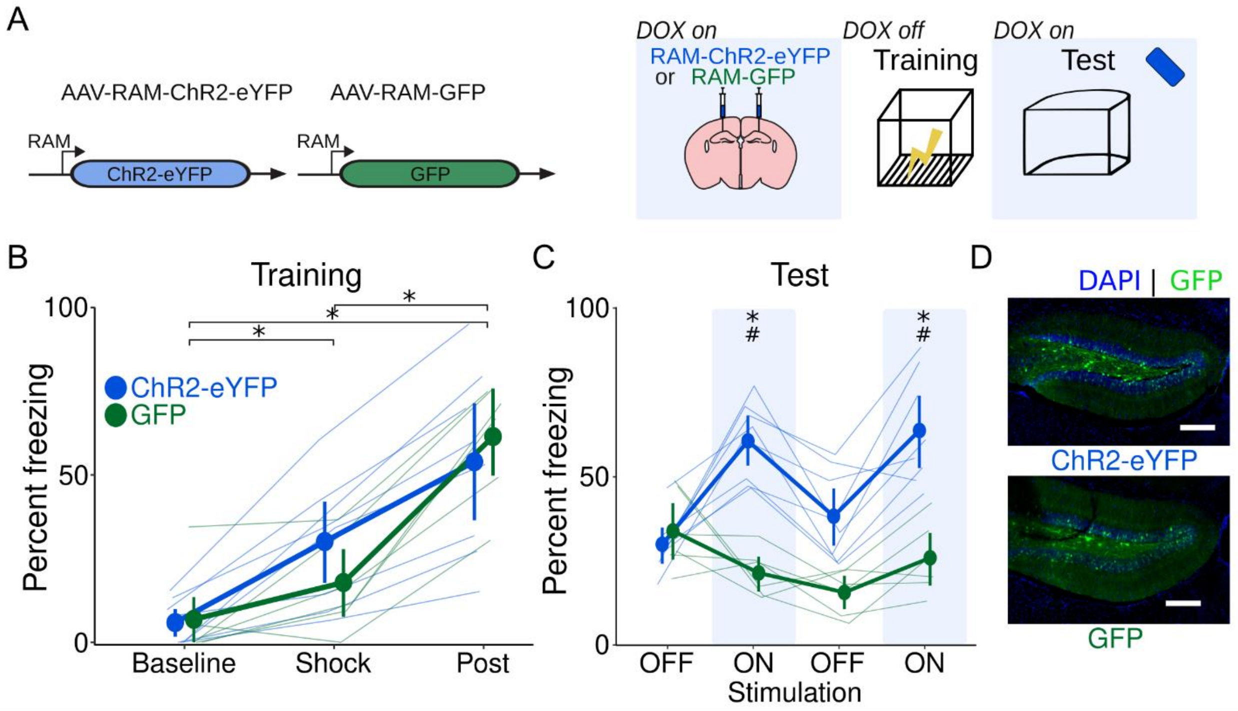

Figure S2. Photostimulation of dentate gyrus ensembles tagged during context fear conditioning is sufficient to induce freezing in novel context in food restricted mice. (**A**) AAV-RAM-ChR2-eYFP or AAV-RAM-GFP viral vectors were microinjected into the dentate gyrus. After food restriction to 90% of free-feed body weight, under DOX diet (200 mg/kg), DOX was removed from diet 48 h before contextual fear conditioning. Mice were replaced on DOX diet and tested in a novel context with light OFF or ON. (**B**) Mean (± 95% confidence interval (CI)) percentage time spent freezing during training, before shock (baseline), during shock and post-shock.*indicates statistical differences between training blocks (*N* = 6-8 mice per group). (**C**) Mean (± 95% CI) percentage time spent freezing in novel context during photostimulation OFF and ON blocks (3 min each). *indicates stimulation block differences whereas #indicates group differences (*N* = 6-8 mice per group). (**D**) Dentate gyrus neurons expressing GFP and ChR2-GFP. Scale bar = 100 μm.

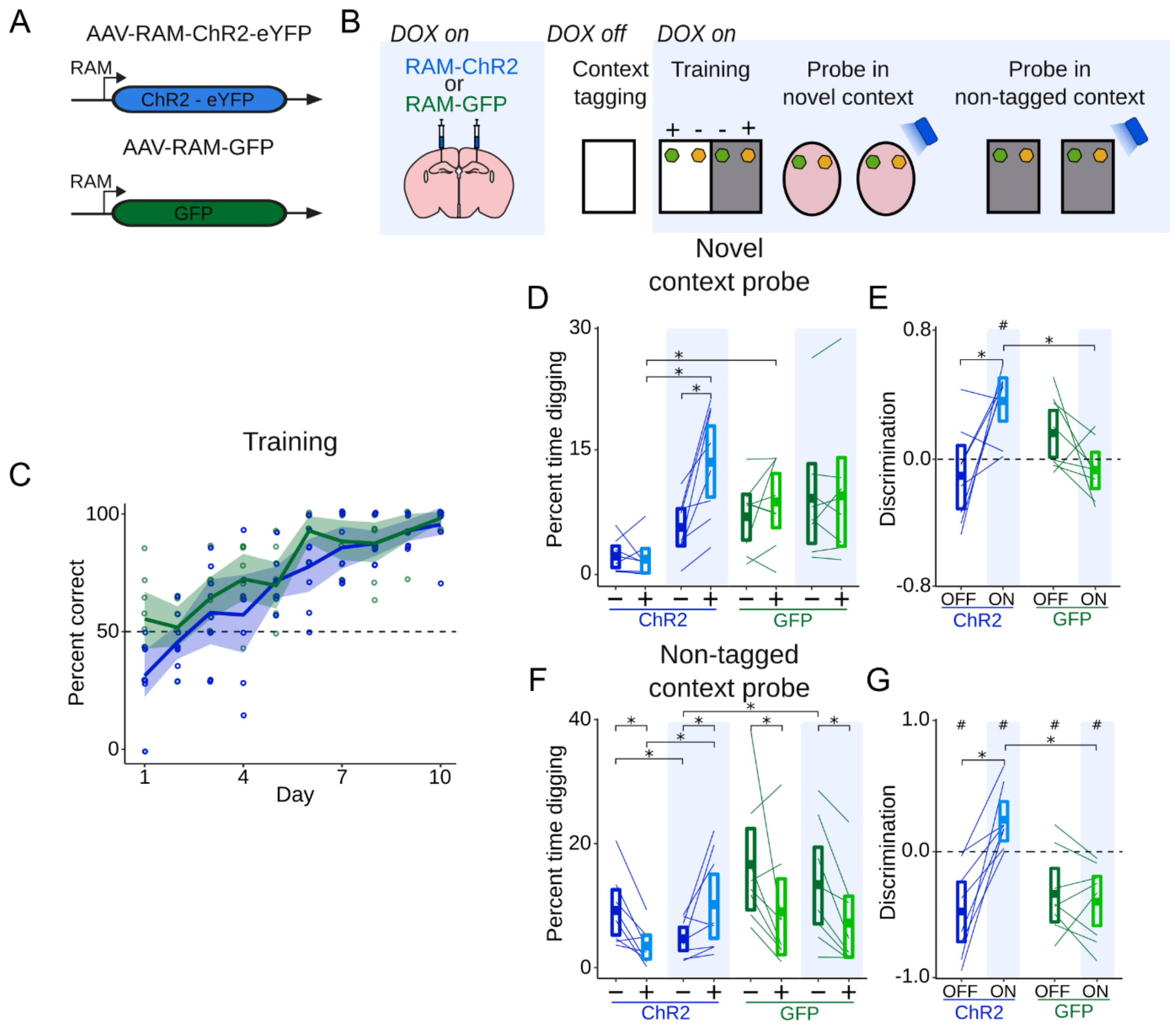

Figure S3. Photostimulation of dentate gyrus ensembles tagged during context habituation session is sufficient to bias memory retrieval in both novel context and non-tagged context. (**A**) Either AAV-RAM-ChR2-eYFP or AAV-RAM-GFP viral vectors were microinjected into the dentate gyrus (**B**). Before training, mice were removed from DOX to tag dentate gyrus ensembles active during placement in novel 'tagged' context. DOX diet was resumed and mice were trained for 10 d, before testing in novel and non-tagged context, with either light ON or OFF. (**C**) Performance improved over training days equivalently in both groups. (Shading represents 95% CI, dashed line = chance performance) (N = 7-8 mice per group). (**D**) Percent time digging and (**E**) discrimination score in probe test in the novel context (box represents 95% CI, dashed line = chance performance). (**F**) Percent time digging and (**G**) discrimination score in probe test in the non-tagged context (box represents 95% CI, dashed line = chance performance).

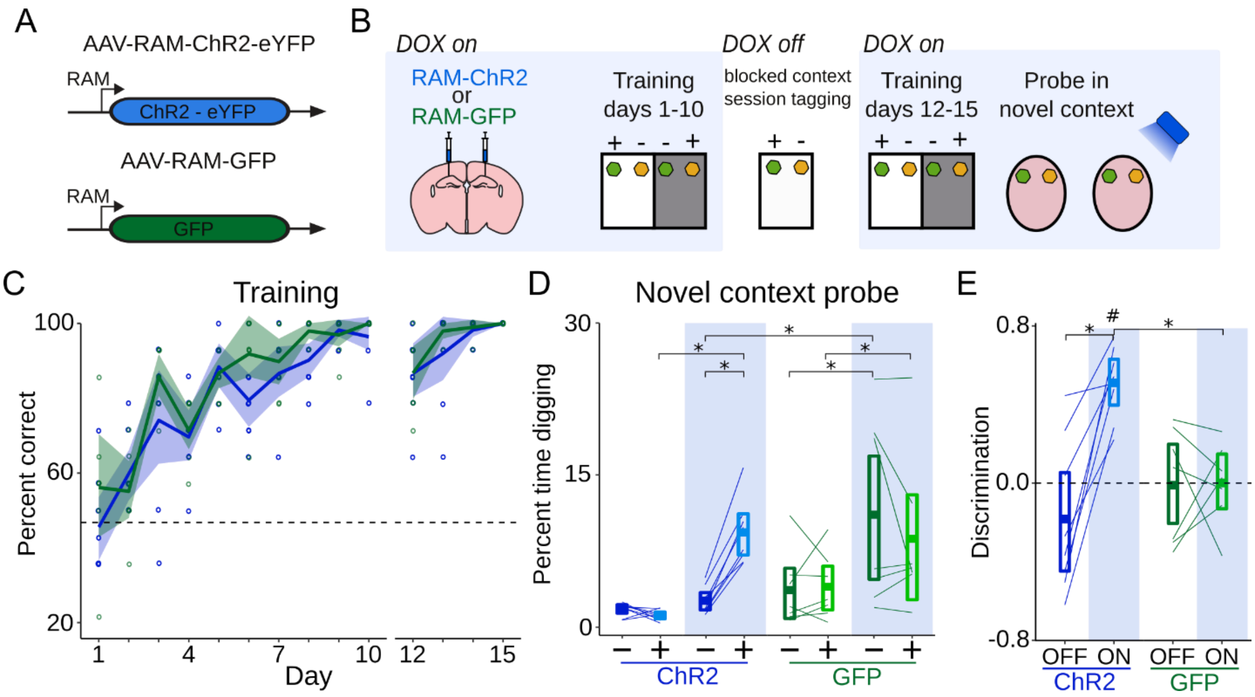

Figure S4. Photostimulation of dentate gyrus ensembles tagged during a blocked context session is sufficient to bias memory retrieval. (**A**) AAV-RAM-ChR2-eYFP or AAV-RAM-GFP viral vectors were microinjected into the dentate gyrus (**B**). Mice were trained for 10 d on DOX. After training, mice were removed from DOX diet to tag dentate gyrus ensembles active during a single blocked ('tagged') context session. Mice were replaced on DOX diet and trained in another blocked context session (in the non-tagged context) and two interleaved context sessions. Mice were tested in a novel context with (ON) or without (OFF) photostimulation. (**C**) Performance increased over training days equivalently in both groups. (Shading represents 95% CI, dashed line = chance performance) (*N* = 7-8 mice per group). (**D**) Percent time digging and (**E**) discrimination score in probe test in the novel context (box represents 95% CI, dashed line = chance performance).

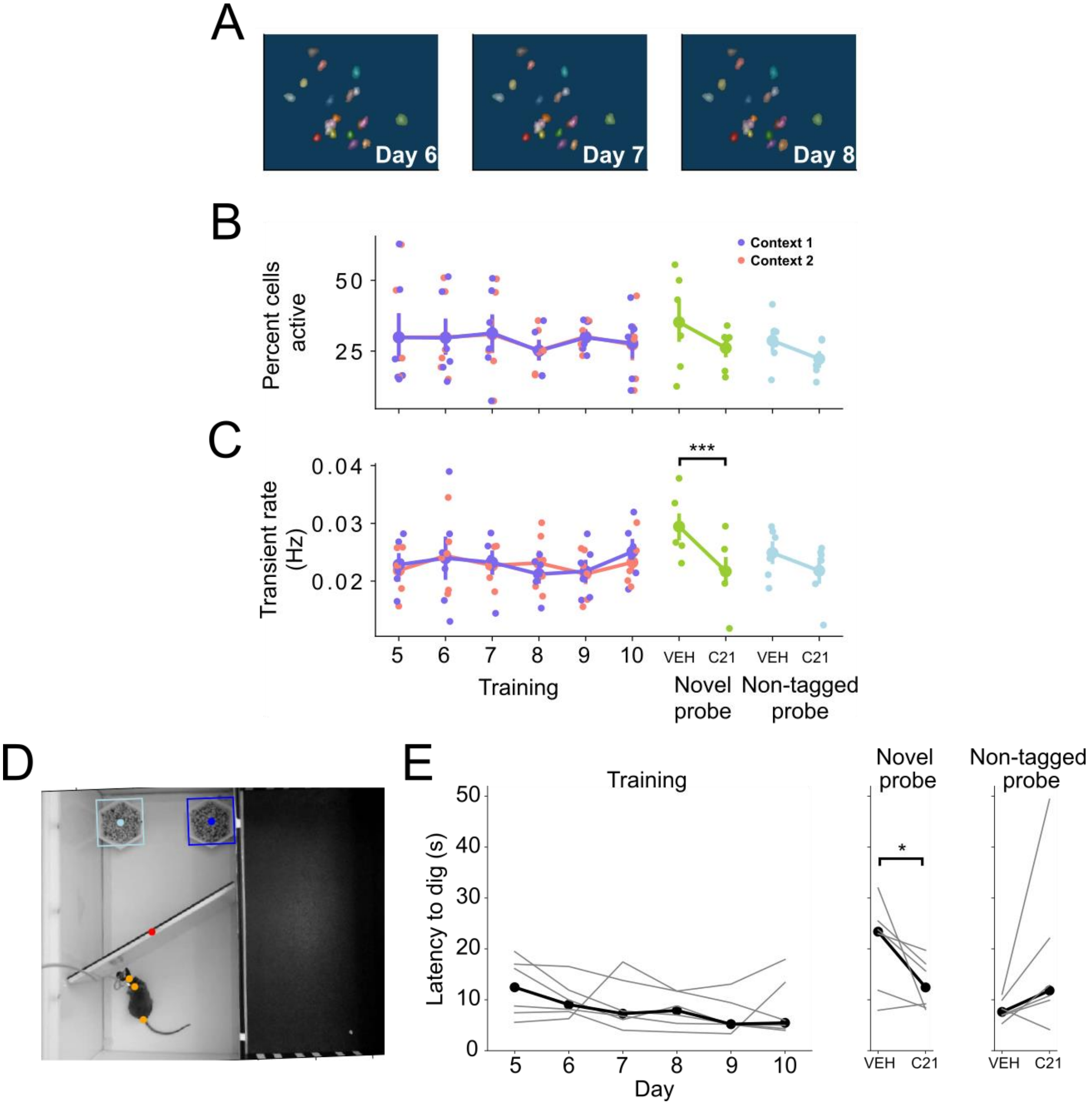

Figure S5. Reactivation of dentate gyrus ensembles tagged during context exposure regulate context-specific patterns of activity in CA1 (additional analyses). (**A**) Example of randomly selected cells that were registered across three consecutive days. Each cell footprint is colored by cell identity. (**B**) Number of cells detected across training and probe sessions ($N = 6$ mice). (**C**) Average transient rate of cells across training and probe sessions ($N = 6$ mice). (**D**) Example frame of DeepLabCut tracking mouse during task (mouse and environment landmarks indicated). (**E**) Latency to dig across training and probe sessions ($N = 6$ mice). Light gray lines indicate latency to first dig (in any reward well) for each individual mouse. Black dots indicate the median value across mice.

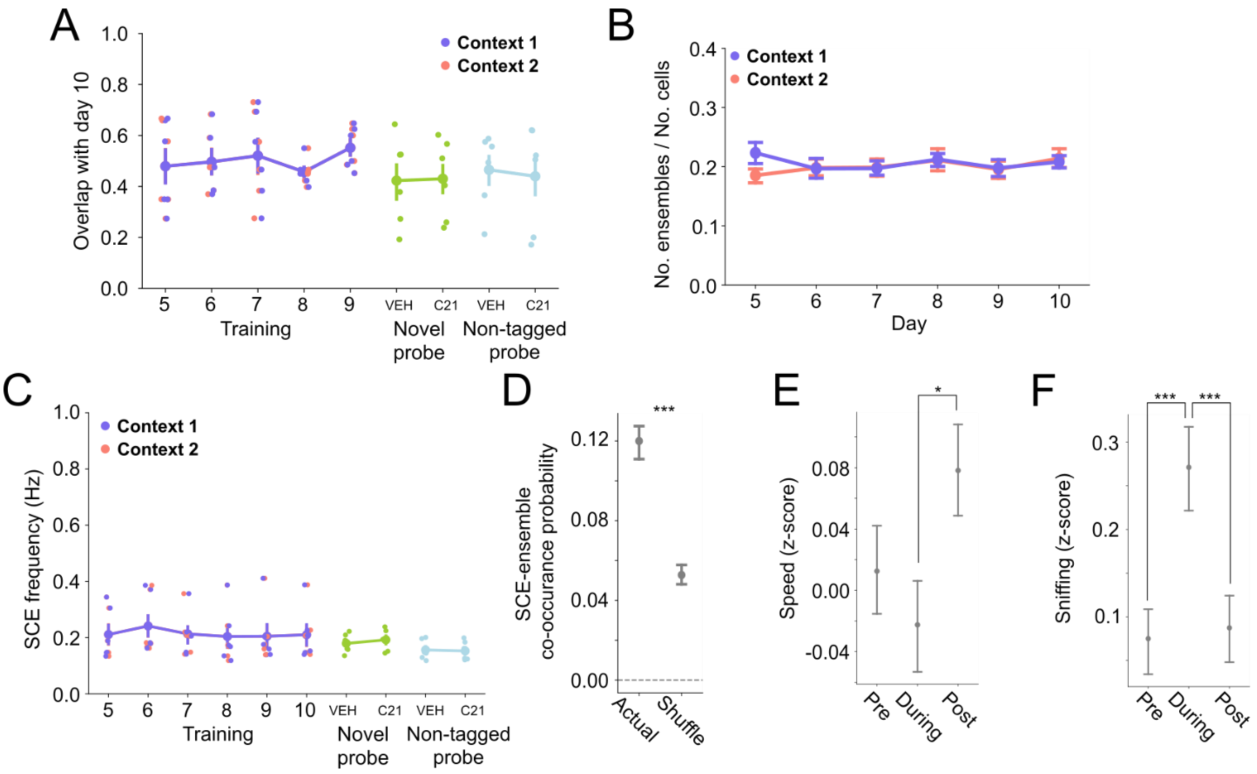

Figure S6. Calcium imaging (additional ensemble analyses). (**A**) Neuronal overlap for each session across training and probe sessions. Overlap was calculated as (No. Cells in Session X and Session 10) / (No. Cells in Session X) (N = 6 mice). (**B**) Number of identified ensembles does not differ between contexts. For each training session and mouse, ensembles were extracted as described in Methods. The ratio between the number of identified ensembles and the number of detected cells is plotted, and colored by context (n = 36 sessions, N = 6 mice). (**C**) Frequency of significant SCEs across training and probe sessions. Timebins exhibiting significant SCEs were calculated as in supplemental methods (N = 6 mice). (**D**) Ensemble activation events are associated with SCEs. Co-occurrence probability between ensemble activation events and SCEs across time was significantly higher than chance level (n = 36 sessions, N = 6 mice). (**E**) Quantification of Fig. 5h. Relative speed was binned (-2 s to -1 s: Pre, -0.5 s to 0.5 s: During, 1 s to 2 s: Post) and averaged. Significant decreases in locomotion were observed during SCEs (n = 36 sessions, N = 6 mice). (**F**) Quantification of Fig. 5i. Relative probability of sniffing was binned (-2 s to -1 s: Pre, -0.5 s to 0.5 s: During, 1s to 2s: Post) and averaged. Significant increase in sniffing behavior was observed during SCEs (n = 36 sessions, N = 6 mice).

REFERENCES AND NOTES

1. E. Tulving, Ecphoric processes in episodic memory. *Philos. Trans. R. Soc. Lond. B. Biol. Sci.i* **302**, 361–371 (1983).

2. E. Tulving, *Elements of Episodic Memory* (Oxford Univ. Press, 1983).

3. R. W. Semon, *The mneme* (G. Allen & Unwin Limited, 1921).

4. P. W. Frankland, S. A. Josselyn, S. Kohler, The neurobiological foundation of memory retrieval. *Nat. Neurosci.* **22**, 1576–1585 (2019).

5. J. M. Gardiner, F. I. Craik, J. Birtwistle, Retrieval cues and release from proactive inhibition. *J. Verbal Learn Verbal Behav.* **11**, 778–783 (1972).

6. O. C. Watkins, M. J. Watkins, Buildup of proactive inhibition as a cue-overload effect. *J. Exp. Psychol. Hum. Learn. Mem.* **1**, 442–452 (1975).

7. D. R. Godden, A. D. Baddeley, Context-dependent memory in two natural environments: On land and underwater. *Br. J. Psychol.* **66**, 325–331 (1975).

8. J. H. Jung, Y. Wang, A. J. Mocle, T. Zhang, S. Kohler, P. W. Frankland, S. A. Josselyn, Examining the engram encoding specificity hypothesis in mice. *Neuron* **111**, 1830–1845.e5 (2023).

9. S. Maren, K. L. Phan, I. Liberzon, The contextual brain: Implications for fear conditioning, extinction and psychopathology. *Nat. Rev. Neurosci.* **14**, 417–428 (2013).

10. E. Tulving, D. M. Thomson, Encoding specificity and retrieval processes in episodic memory. *Psychol. Rev.* **80**, 352–373 (1973).

11. S. M. Smith, E. Vela, Environmental context-dependent memory: A review and meta-analysis. *Psychon. Bull. Rev.* **8**, 203–220 (2001).

12. J. B. Julian, C. F. Doeller, Remapping and realignment in the human hippocampal formation predict context-dependent spatial behavior. *Nat. Neurosci.* **24**, 863–872 (2021).

13. S. M. Polyn, V. S. Natu, J. D. Cohen, K. A. Norman, Category-specific cortical activity precedes retrieval during memory search. *Science* **310**, 1963–1966 (2005).

14. Y. Zhao, B. A. Kuhl, in *Oxford Handbook of Human Memory*, M. J. Kahana, A. D. Wagner, Eds. (Oxford Univ. Press, 2022).

15. T. Staudigl, C. Vollmar, S. Noachtar, S. Hanslmayr, Temporal-pattern similarity analysis reveals the beneficial and detrimental effects of context reinstatement on human memory. *J. Neurosci.* **35**, 5373–5384 (2015).

16. J. S. Nairne, The myth of the encoding-retrieval match. *Memory* **10**, 389–395 (2002).

17. J. R. Epp, R. Silva Mera, S. Kohler, S. A. Josselyn, P. W. Frankland, Neurogenesis-mediated forgetting minimizes proactive interference. *Nat. Commun.* **7**, 10838 (2016).

18. T. Rajji, D. Chapman, H. Eichenbaum, R. Greene, The role of CA3 hippocampal NMDA receptors in paired associate learning. *J. Neurosci.* **26**, 908–915 (2006).

19. A. B. Tort, R. W. Komorowski, J. R. Manns, N. J. Kopell, H. Eichenbaum, Theta-gamma coupling increases during the learning of item-context associations. *Proc. Natl. Acad. Sci. U.S.A.* **106**, 20942–20947 (2009).

20. P. C. Holland, M. E. Bouton, Hippocampus and context in classical conditioning. *Curr. Opin. Neurobiol.* **9**, 195–202 (1999).

21. X. Liu, S. Ramirez, P. T. Pang, C. B. Puryear, A. Govindarajan, K. Deisseroth, S. Tonegawa, Optogenetic stimulation of a hippocampal engram activates fear memory recall. *Nature* **484**, 381–385 (2012).

22. A. P. Maurer, L. Nadel, The continuity of context: A role for the hippocampus. *Trends Cogn. Sci.* **25**, 187–199 (2021).

23. S. Ramirez, X. Liu, P. A. Lin, J. Suh, M. Pignatelli, R. L. Redondo, T. J. Ryan, S. Tonegawa, Creating a false memory in the hippocampus. *Science* **341**, 387–391 (2013).

24. M. E. Hasselmo, H. Eichenbaum, Hippocampal mechanisms for the context-dependent retrieval of episodes. *Neural Netw.* **18**, 1172–1190 (2005).

25. R. Hirsh, The hippocampus and contextual retrieval of information from memory: A theory. *Behav. Biol.* **12**, 421–444 (1974).

26. C. Ranganath, Binding items and contexts: The cognitive neuroscience of episodic memory. *Curr. Dir. Psychol. Sci.* **19**, 131–137 (2010).

27. J. B. Julian, C. F. A. Doeller, Context in spatial and episodic memory, in *The Cognitive Neurosciences* (MIT Press, 2020), pp. 217-232.

28. A. T. Sørensen, Y. A. Cooper, M. V. Baratta, F. J. Weng, Y. Zhang, K. Ramamoorthi, R. Fropf, E. LaVerriere, J. Xue, A. Young, C. Schneider, C. R. Gøtzsche, M. Hemberg, J. C. Yin, S. F. Maier, Y. Lin, A robust activity marking system for exploring active neuronal ensembles. *eLife* **5**, e13918 (2016).

29. A. Keresztes, C. T. Ngo, U. Lindenberger, M. Werkle-Bergner, N. S. Newcombe, Hippocampal maturation drives memory from generalization to specificity. *Trends Cogn. Sci.* **22**, 676–686 (2018).

30. A. D. Jacob, A. I. Ramsaran, A. J. Mocle, L. M. Tran, C. Yan, P. W. Frankland, S. A. Josselyn, A compact head-mounted endoscope for in vivo calcium imaging in freely behaving mice. *Curr. Protoc. Neurosci.* **84**, e51 (2018).

31. J. S. Biane, M. A. Ladow, F. Stefanini, S. P. Boddu, A. Fan, S. Hassan, N. Dundar, D. L. Apodaca-Montano, L. Z. Zhou, V. Fayner, N. I. Woods, M. A. Kheirbek, Neural dynamics underlying associative learning in the dorsal and ventral hippocampus. *Nat. Neurosci.* **26**, 798–809 (2023).

32. E. Mizrak, N. R. Bouffard, L. A. Libby, E. D. Boorman, C. Ranganath, The hippocampus and orbitofrontal cortex jointly represent task structure during memory-guided decision making. *Cell Rep.* **37**, 110065 (2021).

33. S. Reinert, M. Hubener, T. Bonhoeffer, P. M. Goltstein, Mouse prefrontal cortex represents learned rules for categorization. *Nature* **593**, 411–417 (2021).

34. E. H. Nieh, M. Schottdorf, N. W. Freeman, R. J. Low, S. Lewallen, S. A. Koay, L. Pinto, J. L. Gauthier, C. D. Brody, D. W. Tank, Geometry of abstract learned knowledge in the hippocampus. *Nature* **595**, 80–84 (2021).

35. J. P. Cunningham, B. M. Yu, Dimensionality reduction for large-scale neural recordings. *Nat. Neurosci.* **17**, 1500–1509 (2014).

36. A. Rubin, L. Sheintuch, N. Brande-Eilat, O. Pinchasof, Y. Rechavi, N. Geva, Y. Ziv, Revealing neural correlates of behavior without behavioral measurements. *Nat. Commun.* **10**, 4745 (2019).

37. L. Meshulam, J. L. Gauthier, C. D. Brody, D. W. Tank, W. Bialek, Collective behavior of place and non-place neurons in the hippocampal network. *Neuron* **96**, 1178–1191.e4 (2017).

38. S. Panzeri, M. Moroni, H. Safaai, C. D. Harvey, The structures and functions of correlations in neural population codes. *Nat. Rev. Neurosci.* **23**, 551–567 (2022).

39. R. E. Hampson, D. R. Byrd, J. K. Konstantopoulos, T. Bunn, S. A. Deadwyler, Hippocampal place fields: Relationship between degree of field overlap and cross-correlations within ensembles of hippocampal neurons. *Hippocampus* **6**, 281–293 (1996).

40. A. D. Grosmark, F. T. Sparks, M. J. Davis, A. Losonczy, Reactivation predicts the consolidation of unbiased long-term cognitive maps. *Nat. Neurosci.* **24**, 1574–1585 (2021).

41. A. Malvache, S. Reichinnek, V. Villette, C. Haimerl, R. Cossart, Awake hippocampal reactivations project onto orthogonal neuronal assemblies. *Science* **353**, 1280–1283 (2016).

42. H. R. Joo, L. M. Frank, The hippocampal sharp wave-ripple in memory retrieval for immediate use and consolidation. *Nat. Rev. Neurosci.* **19**, 744–757 (2018).

43. B. E. Pfeiffer, D. J. Foster, Hippocampal place-cell sequences depict future paths to remembered goals. *Nature* **497**, 74–79 (2013).

44. C. T. Wu, D. Haggerty, C. Kemere, D. Ji, Hippocampal awake replay in fear memory retrieval. *Nat. Neurosci.* **20**, 571–580 (2017).

45. Y. Norman, E. M. Yeagle, S. Khuvis, M. Harel, A. D. Mehta, R. Malach, Hippocampal sharp-wave ripples linked to visual episodic recollection in humans. *Science* **365**, eaax1030 (2019).

46. J. J. Sakon, M. J. Kahana, Hippocampal ripples signal contextually mediated episodic recall. *Proc. Natl. Acad. Sci. U.S.A.* **119**, e2201657119 (2022).

47. A. P. Vaz, S. K. Inati, N. Brunel, K. A. Zaghloul, Coupled ripple oscillations between the medial temporal lobe and neocortex retrieve human memory. *Science* **363**, 975–978 (2019).

48. M. F. Carr, S. P. Jadhav, L. M. Frank, Hippocampal replay in the awake state: A potential substrate for memory consolidation and retrieval. *Nat. Neurosci.* **14**, 147–153 (2011).

49. A. D. Redish, Vicarious trial and error. *Nat. Rev. Neurosci.* **17**, 147–159 (2016).

50. S. A. Josselyn, S. Kohler, P. W. Frankland, Finding the engram. *Nat. Rev. Neurosci.* **16**, 521–534 (2015).

51. C. Jou, J. R. Hurtado, S. Carrillo-Segura, E. H. PARK, A. A. Fenton, On the results of causal optogenetic engram manipulations. bioRxiv 2023.05.15.540888 (2023). https://doi.org/10.1101/2023.05.15.540888.

52. T. Sasaki, V. C. Piatti, E. Hwaun, S. Ahmadi, J. E. Lisman, S. Leutgeb, J. K. Leutgeb, Dentate network activity is necessary for spatial working memory by supporting CA3 sharp-

wave ripple generation and prospective firing of CA3 neurons. *Nat. Neurosci.* **21**, 258–269 (2018).

53. T. Nakashiba, D. L. Buhl, T. J. McHugh, S. Tonegawa, Hippocampal CA3 output is crucial for ripple-associated reactivation and consolidation of memory. *Neuron* **62**, 781–787 (2009).

54. A. Ylinen, A. Bragin, Z. Nadasdy, G. Jando, I. Szabo, A. Sik, G. Buzsaki, Sharp wave-associated high-frequency oscillation (200 Hz) in the intact hippocampus: Network and intracellular mechanisms. *J. Neurosci.* **15**, 30–46 (1995).

55. A. Hupbach, O. Hardt, R. Gomez, L. Nadel, The dynamics of memory: Context-dependent updating. *Learn. Mem.* **15**, 574–579 (2008).

56. A. P. Yonelinas, C. Ranganath, A. D. Ekstrom, B. J. Wiltgen, A contextual binding theory of episodic memory: Systems consolidation reconsidered. *Nat. Rev. Neurosci.* **20**, 364–375 (2019).

57. T. D. Goode, K. Z. Tanaka, A. Sahay, T. J. McHugh, An integrated index: Engrams, place cells, and hippocampal memory. *Neuron* **107**, 805–820 (2020).

58. T. J. Teyler, P. DiScenna, The hippocampal memory indexing theory. *Behav. Neurosci.* **100**, 147–154 (1986).

59. T. Hainmueller, M. Bartos, Parallel emergence of stable and dynamic memory engrams in the hippocampus. *Nature* **558**, 292–296 (2018).

60. E. Eich, Searching for mood dependent memory. *Psychol. Sci.* **6**, 67–75 (1995).

61. C. G. Ucros, Mood state-dependent memory: A meta-analysis. *Cognit. Emot.* **3**, 139–169 (1989).

62. M. W. Howard, M. J. Kahana, A distributed representation of temporal context. *J. Math. Psychol.* **46**, 269–299 (2002).

63. H. Dana, T. W. Chen, A. Hu, B. C. Shields, C. Guo, L. L. Looger, D. S. Kim, K. Svoboda, Thy1-GCaMP6 transgenic mice for neuronal population imaging in vivo. *PLOS ONE* **9**, e108697 (2014).

64. R. C. Bolles, Species-specific defense reactions and avoidance learning. *Psychol. Rev.* **77**, 32–48 (1970).

65. A. Mathis, P. Mamidanna, K. M. Cury, T. Abe, V. N. Murthy, M. W. Mathis, M. Bethge, DeepLabCut: Markerless pose estimation of user-defined body parts with deep learning. *Nat. Neurosci.* **21**, 1281–1289 (2018).

66. P. Zhou, S. L. Resendez, J. Rodriguez-Romaguera, J. C. Jimenez, S. Q. Neufeld, A. Giovannucci, J. Friedrich, E. A. Pnevmatikakis, G. D. Stuber, R. Hen, M. A. Kheirbek, B. L. Sabatini, R. E. Kass, L. Paninski, Efficient and accurate extraction of in vivo calcium signals from microendoscopic video data. *eLife* **7**, e28728 (2018).

67. L. Sheintuch, A. Rubin, N. Brande-Eilat, N. Geva, N. Sadeh, O. Pinchasof, Y. Ziv, Tracking the same neurons across multiple days in Ca^{2+} imaging data. *Cell Rep.* **21**, 1102–1115 (2017).

68. J. Friedrich, P. Zhou, L. Paninski, Fast online deconvolution of calcium imaging data. *PLOS Comput. Biol.* **13**, e1005423 (2017).

69. F. Pedregosa, G. Varoquaux, A. Gramfort, V. Michel, B. Thirion, O. Grosel, M. Nlondel, P. Prettenhofer, R. Weiss, V. Dubourg, J. Vanderplas, A. Passos, D. Cournapeau, M. Brucher, M. Perrot, E. Duchesnay, Scikit-learn: Machine learning in Python. *J. Mach. Learn. Res.* **12**, 2825–2830 (2011).

70. H. Wickham, R. François, L. Henry and K. Müller, A Grammar of Data Manipulation [R package dplyr version 1.0.2] (2021).

71. D. Bates, M. Mächler, B. Bolker, S. Walker, Fitting linear mixed-effects models using lme4. *J. Stat. Softw.* **67**, 1–48 (2015).

72. A. Kuznetsova, P. B. Brockhoff, R. H. Christensen, lmerTest package: Tests in linear mixed effects models. *J. Stat. Softw.* **82**, 1–26 (2017).

73. R. Lenth, emmeans: Estimated Margin2l Means, aka Least-Squares Means. R package version 1.10.3-090003, https://rvlenth.github.io/emmeans/, https://rvlenth.github.io/emmeans/ (2024).

74. H. Wickham, Programming with ggplot2, in *Ggplot2: Elegant graphics for data analysis* (Springer, 2016), pp. 241–253.

75. A. Kassambara, Package ‘ggpubr’, R package version 0.1 6, GitHub (2020).
